# Additive Genetic Behavior of Stem Solidness in Wheat (*Triticum aestivum* L.)

**DOI:** 10.1038/s41598-020-64470-x

**Published:** 2020-04-30

**Authors:** Naresh Kumar Bainsla, Rajbir Yadav, Gyanendra Pratap Singh, Ram Kumar Sharma

**Affiliations:** 1 Division of Genetics, ICAR- Indian Agricultural Research Institute, New Delhi, 110012 India; 2 Indian Institute of Wheat and Barley Research, Karnal, 132001 India

**Keywords:** Plant breeding, Plant genetics

## Abstract

Stem solidness in wheat is an important architectural trait to support the erect behavior of the plant. The varieties with high yield potential due to increased sink strength tend to lodge either because of poor anchorage or weak stem. The solid stem can partially counter the tradeoff between biomass driven yield gain irrespective of the plant height. Stem solidness being a complex trait with highly variable expressivity, understanding its genetic behavior in different genetic backgrounds is highly essential to integrate this trait in the breeding program. In this study, the expressivity of a solid stem in different internodes was investigated in nine F_2_ populations selected from 34 F_1_s (solid stem × hollow stem and hollow stem × hollow stem). The progeny of solid stem type F_1_ plants from hollow stem parents indicated the complementation of favorable alleles dispersed among the parents. Non-confirmation to digenic complementary (9:7) model of inheritance and polynomial distribution of the trait in all F_2_ populations indicates multiple factors complementation in the additive fashion for stem solidness.

## Introduction

Wheat is the principal staple food of the world with a sizeable contribution from India, is the most important crop for global food supply. The North Western Plain Zone (NWPZ) of India is optimal for wheat production due to the accessibility of supplemented irrigation and good management practices^1^. However, declining yield gain in bread wheat breeding poses a big challenge for meeting the requirement of the populous country by 2050, particularly under the constraints of increased competition for the cropped area, depleting water resources and changing climate^2^. The harvest index (HI) under the optimum plant population and the best management conditions^3^ is already optimized at about 0.45 and a further increase in HI will require a complete change in plant architecture. An increase in plant biomass can still be explored for furthering the yield gain in the future. However, increased biomass will further the risk of lodging unless we bring about structural changes both in stems and roots. The abrupt increase of temperature in the desert of Rajasthan and snowfall events in lower and Himalayan states brings tropospheric changes resulting in unwarranted rain and or storm generally between the second fortnight of January - middle of March causing large scale lodging in the wheat crop in grain filling stage^2^. The uncertainty in crop production regime due to erratic environmental conditions poses a serious threat to further realization of high productivity genotypes due to wheat vulnerability to lodging^2,3^. The lodging in wheat can occur both in the stem as well as the root level. A weaker stem or tiller will bend permanently due to wind force exerted on plant compared to a stiffer and broader diameter stem which will resonate along with the wind and will come to its original position sooner owing to its better elasticity^4–6^. The force exerted on the stem will pass the turning moment to the root zone and a weaker root system will fail to cause the root lodging. To support each 10 cm increase in height, the diameter of the bottom internode and the root plate spread must be increased by 0.36 and 3.7 mm, respectively^7^. Therefore, integration of stem solidness especially in lower internodes with better root plate seems to be a plausible option to relatively meet the requirement of biomass and the requisite strength to hold heavier spikes^4,6^. Stem solidness confers resistance to wheat stem sawfly and has been suggested as an important component for conferring lodging tolerance^6,8,9^.

The stem solidness has been reported as a simply inherited genetic trait controlled primarily by major gene(s) with various levels of expressivity. There are different reports regarding the inheritance of the solid-stem trait ranging from one major gene with several minor, modifying genes to four genes, with one being epistatic to the others, based on two solid- stem × hollow-stem crosses^10,11^. A similar study based on monosomic analysis of solid-stem reported that at least five chromosomes carried genes promoting pith production and at least three chromosomes carried genes inhibiting pith production^12^. A significant solid-stem × hollow-stem parent interaction was observed for stem solidness score suggesting epistatic gene action in the inheritance of this trait^13^. The trait has also been reported to be negatively correlated with yield in older reports^14,15^; however, later studies confirmed no such association of genomic regions explaining the variation of solid stem trait with decreasing the yield potential^16^. Moreover, the development of high yielding, solid-stem cultivars are not limited by undesirable associations between degree of stem solidness and other agronomic traits^2,13,17–20^.

Besides, cross combinations of solid versus hollow stem parents, there are no reports of getting solid stem F_1_ plants from hollow stem parents. Moreover, most of the past genetic studies are based on a single bi-parental population implicating to limited genetic background. To address those shortcomings of the past studies, a comprehensive genetic study was carried out in multiple genetic backgrounds comprising of nine F_2_ populations (as described in Materials and Methods section) which included both the hollow × hollow and solid × hollow stem type of cross combinations to understand the expressivity of stem solidness in different genetic combinations, to find out better parental combinations and superior recombinants from the progenies. Additionally, the frequency of superior recombinants with at least three internodes of stem solidness and frequency of spikes with more than twenty spikelets were also determined with the aim that stem solidness can be favorably used for the development of high yield potential genotypes.

## Material and Methods

The field experiments were carried out at the experiment station, located at 28°38′20′′ N and 77°9′E, of Indian Agricultural Research Institute (ICAR), New Delhi, India. The soil type is a moderately alkaline sandy loam with pH = 7.5–8.5 and electrical conductivity ranging from 0.4 to 0.6 dS/m. Moreover, the organic carbon content was low (0.41%), and the available Nitrogen (N), Phosphorus (P) and Potassium (K) content at 0–15 cm depth were <280, 25–30, and 120–280 kg/ha, respectively. The recommended dose of NPK fertilizer 150: 60: 40 kg/ha was applied to ensure optimum crop growth. For all the field experiments, the sowing was done during the third week of November, and standard agronomic management practices were followed with five supplementary irrigations scheduled according to the growth stage of the crop. A total of 413 cross combinations were made during 2015–16 in winter season with about 10 spikes per combination, and the F_1_ seeds were sown during 2016–17 growing season in 0.5 m long and 0.25 wide paired rows where seeds were sown at 5 cm apart in order give enough space for each plant for optimal growth. At least 10 representative tillers from each of the 413 crosses were evaluated for the stem solidness trait. Out of total F1 combinations, 34 expressed solid stem (Table 1), 106 were semisolid, 24 were necrotic and remaining were hollow-stem type. The F_2_ populations were grown in 2017–18 where the seeds were space sown at 10 cm apart keeping the family size of 400 to 500 in each population.

**Table 1:** Parental combinations resulting in solid stem expression in F_1_ plants.

S. No.	Parent 1	Parent 2	Cross-type
1	**HD2932**	**HD2329**	Hollow × Solid
2	**HD2932**	HUW666	Hollow × Hollow
3	**HD2932**	MP4010	Hollow × Hollow
4	WH1105	HD2851/Yr10	Hollow × Hollow
5	WH1105	**HD2329**	Hollow × Solid
6	DPW621–50	**HD2329**	Hollow × Solid
7	PBW373	**HD2329**	Hollow × Solid
8	**HD2967**	JKW230	Hollow × Hollow
9	**HD2967**	HD2894	Hollow × Hollow
10	**HD2967**	RAJ4422	Hollow × Hollow
11	HD3086	HD2987	Hollow × Hollow
12	HD3086	HD2189	Hollow × Hollow
13	PBW677	HD2967	Hollow × Hollow
14	K1204	CL3734	Hollow × Hollow
15	NW6049	**HD2329**	Hollow × Solid
16	NW6049	HD3159	Hollow × Hollow
17	PBW725	PBW707	Hollow × Hollow
18	K1301	HD2851/Yr10	Hollow × Hollow
19	PBW709	NW6029	Hollow × Hollow
20	DBW143	HD2997	Hollow × Hollow
21	HD2833	PBW692	Hollow × Hollow
22	JPT	PBW725	Hollow × Hollow
23	DBW134	NW6029	Hollow × Hollow
24	GW322	HD2733*3//HD2733/HD3016	Hollow × Hollow
25	**HD2733**	HS513	Hollow × Hollow
26	**HD2733**	HS507	Hollow × Hollow
27	**HD2733**	WH1105//HD3059/DBW98	Hollow × Hollow
28	**HD2733**	CL3734//HD2967/HPW228	Hollow × Hollow
29	**HD2733**	WH1105//HD2967/HPW228	Hollow × Hollow
30	**WH1175**	**HD2329**	Hollow × Solid
31	**WH1175**	WH1187	Hollow × Hollow
32	**WH1175**	CL3734/WH1105/PBW677	Hollow × Hollow
33	**WH1175**	HD30	Hollow × Hollow
34	BRW3767	WH1154	Hollow × Hollow

*The bold font genotypes are better combiners for the trait. HD2329 is the solid stemmed genotype.

The solid stem was characterized into three categories based on the expression of pith formation in different internodes (Table 2). The pith formation or the solid tissue deposition was observed on the transverse section of the middle of the internode, and the corresponding stems were classified as solid stem - where pith formation was more than 90% (grade 3), semisolid stem - with 50–90% pith filling (grade 2), and hollow stem - with less than 50% filling (grade 1) based on stem diameter measurements.

**Table 2:** Grades of solidness.

Pith expression level	Grade assigned	Category
<50% filled	1	Hollow
50–90% filled	2	Semi Solid
90–100%	3	Solid

Of the 34 crosses, nine combinations as described in Table 3 were chosen for further phenotyping of stem solidness in individuals of the segregating population. Three representative tillers were taken from random plants from each population, and they were mixed and sorted according to their height representing primary and secondary tillers from each of the population. The observations were recorded on the internode length (cm), the extent of solidness in each internode i.e., solid, semi-solid and hollow, numbers of spikelets/spike and the total length of the tiller (cm).

**Table 3:** The salient features of parents used to study the expression in nine F_2_ populations.

S. No.	Parent 1	Salient features	Parent 2	Salient features
1	HD3086	A hollow stem high yielding variety released in 2014 for cultivation in the northwestern plain zone of India for timely sown and irrigated conditions.	HD2189	A hollow stem variety released in 1980 for cultivation in the peninsular zone of India for timely sown and irrigated conditions.
2			HD2987	A hollow stem variety released in 2011for the Peninsular Zone of India for restricted irrigated conditions.
3	HD2967)	A high yielding mega variety released in 2010 for cultivation in the northwestern plain zone (NWPZ) of India for timely sown and irrigated conditions. It has a hollow stem.	RAJ4422	An improved genetic resource having hollow stem taken from the coordinated trials
4	WH1105	A high yielding variety having a hollow stem released in 2013 for cultivation in the northwestern plain zone of India for timely sown and irrigated conditions.	HD2329	A very popular and mega variety of NWPZ. Released in 1985 for irrigated and timely sown conditions having solidness up to three internodes.
5	DPW621–50	A high yielding variety released in 2011 for cultivation in NWPZ for timely sown and irrigated conditions.		
6	PBW373	A hollow stem variety released in 1997 for cultivation in NWPZ of India for late sown and irrigated conditions.		
7	HD2932	A high yielding variety released in 1997 for cultivation in the central zone for timely sown and irrigated conditions.	HUW666	Improved genetic resource taken from the coordinated trials
8	PBW725	A hollow stem variety released in 2016 for cultivation in Punjab state.	PBW707	An improved genetic resource taken from the coordinated trials
9	K1301	An improved genetic resource taken from the coordinated trials	HD2851/Yr10	An improved genetic stock in the background of HD2851 released for cultivation in Delhi and the national capital region in 2007

The percent of tillers in each of solidness classes representing the expressivity were plotted against different internodes. The regression equation was also established using the best model of regression explaining maximum variability for each class using the plot function in MS Excel. The interclass correlations based on Pearson correlation coefficients were determined by using the *corplot* function along with hierarchical clustering with alpha = 0.05 using the R software package. The variables were standardized before analysis using scale function for data in R software.

The K means clustering using the Hartigan-Wong algorithm was applied for determining the population structure using centroid from the matrix of standardized variables^18^. The total variance explained was plotted against two principal components on the x and y-axes. The optimum number of K clusters was determined by the elbow method wherein the within-cluster sum of squares (WSS) was plotted up to 10 groups, and point after which there is no significant reduction in WSS was taken as the threshold. The R software packages *K means* and *factoextra* were used for summarizing the outputs.

The desirable recombinants could be visualized based on the pair-wise scatter plot of height versus spikelet number, and grade total as an indicator of the solidness was used in a unique color scheme using *the ggplot* function in the *ggplot2* package in R software. In each population, the scale of desirable recombinants could be changed depending upon the frequency of the same. A general criterion could be the identification of plants having a height of over 90 cm, the number of spikelets equal to or more than 20 and a grade of solidness equal to or more than 8, which means at least two internodes are solid and third could be semisolid or solid.

## Results

### Expressivity of solidness

Based on the extensive evaluation of F_1_ plants from 413 different cross combinations, 34 were found to express solidness in lower internodes (Table 1). The 106 combinations were found to have semisolid expression, 249 combinations had hollow stems and 24 combinations expressed necrotic seedlings. The combinations expressing the solid stem in F_1_ indicate the genetic background favorable for expression of the trait. The good combiners for the trait could be HD2329, HD2733, and WH1175 which have shown good expression of the trait in combination with different parents.

**Figure 1:**
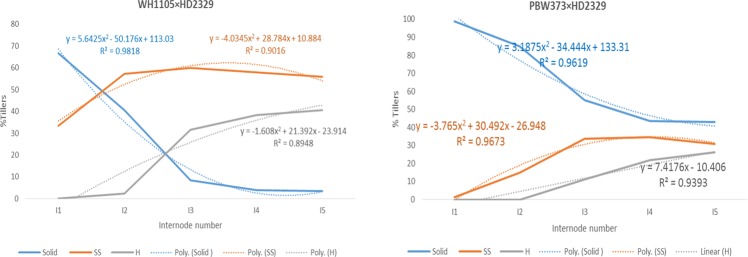
Internode-wise expression of stem solidness in different populations (The y-axis the % tillers expressing the grade of solidness and x-axis shows the internodes starting from bottom to top. The dotted line shows the regression line while the solid line shows the data trend).

Nine F_2_ populations advanced from the bulked ear heads from single F_1_ plants were used for further study (Table 3.). The expression of solid stem character in three grades was expressed as a percent of tillers in each grade plotted against the different internodes as given in (Fig. 1). The similarity of the pattern was observed in the cross combinations (HD3086× HD2189; HD2967× RAJ4422; WH1105× HD2329) wherein the regression lines of solid and semi-solid types intersected before the second internode, therefore, semisolid types being more prominent than the solid type of tillers in population. The best fit regression equations indicated that solid stem trait followed the polynomial distribution of second-order while semisolid and hollow followed the polynomial distribution of second or higher order indicating that interaction of at least two factors favorable for solid trait while three or more factors favored for the hollowness. The complete expression of solidness or pith was observed in 50 to 60 percent tillers in the first internode with little decrease in the second internode to 40% in WH1105 × HD2329 population while sharp decrease to 20% in HD3086 × HD2189 and HD2967 × RAJ4422 combinations and terminating in the third internode. The semisolid behavior was observed in about 50% tillers in the second internode which remained expressed up to 5^th^ internode in WH1105 × HD2329 while terminated in 3^rd^ internode onward in the remaining two populations.

The populations DPW621–50 × HD2329, HD3086 × HD2987, and HD2932 × HUW666 wherein the solid, semisolid and hollow regression lines intersect at second internode. The regression equation showed the binomial distribution for solid and polynomial for semisolid types. There was a comparatively slow decrease in the frequency of solid pith from first to the second internode followed by a sharp decrease in the third internode. The expression of the semi-solid type was maximum in the second internode.

In the population, PBW373 × HD2329 the solidness expressed in comparatively higher (40%) number of tillers in the fifth internode. The solid, semisolid and hollow lines did not intersect in this combination. The other two combinations viz. PBW725 × PBW707 and K1301 × HD2851/Yr10 were having the expression of solid types in 40% tillers in three internodes. All the categories showed binomial distribution except semisolid types in K1301 × HD2851/Yr10 wherein it was polynomial indicating some complex interaction of factors.

**Figure 2:**
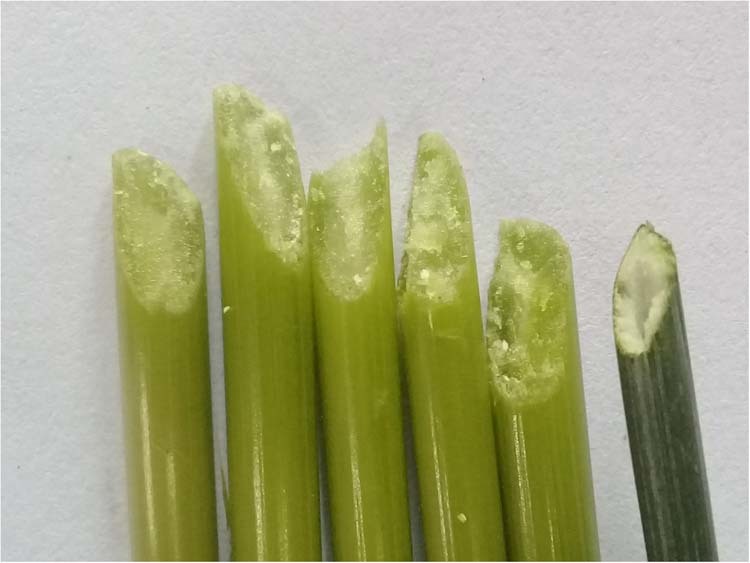
Slanting cut of different internodes from the single tiller.

**Figure 3:**
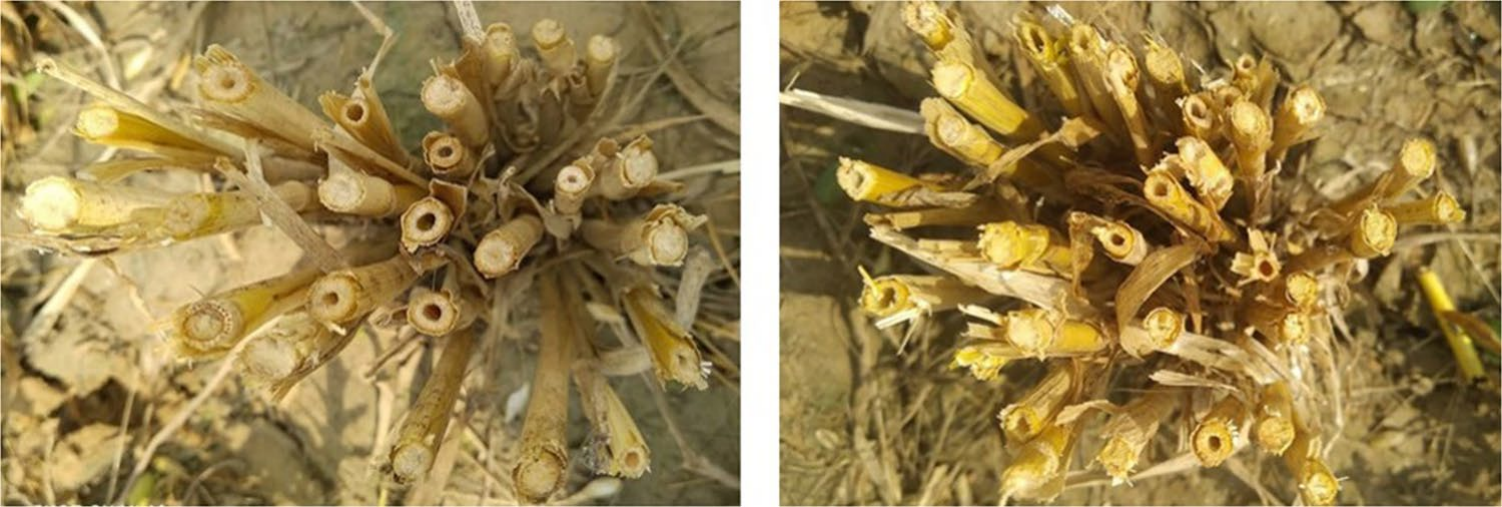
Different tillers of a plant having variable expressivity of solidness.

The fact that HD2329 was the only parent with a solid stem phenotype could combine differentially with other genotypes resulting in the expression of all three categories of solidness. This indicates the absence of dominant type gene action and confirms the presence of complementary gene interaction. The solidness expressed variably even within a plant as all the internodes within a tiller vary for solidness (Fig. 2) as well as all the tillers from the same plant are not solid (Fig. 3).

### Discriminant analysis of populations based on K-means

**Figure 4:**
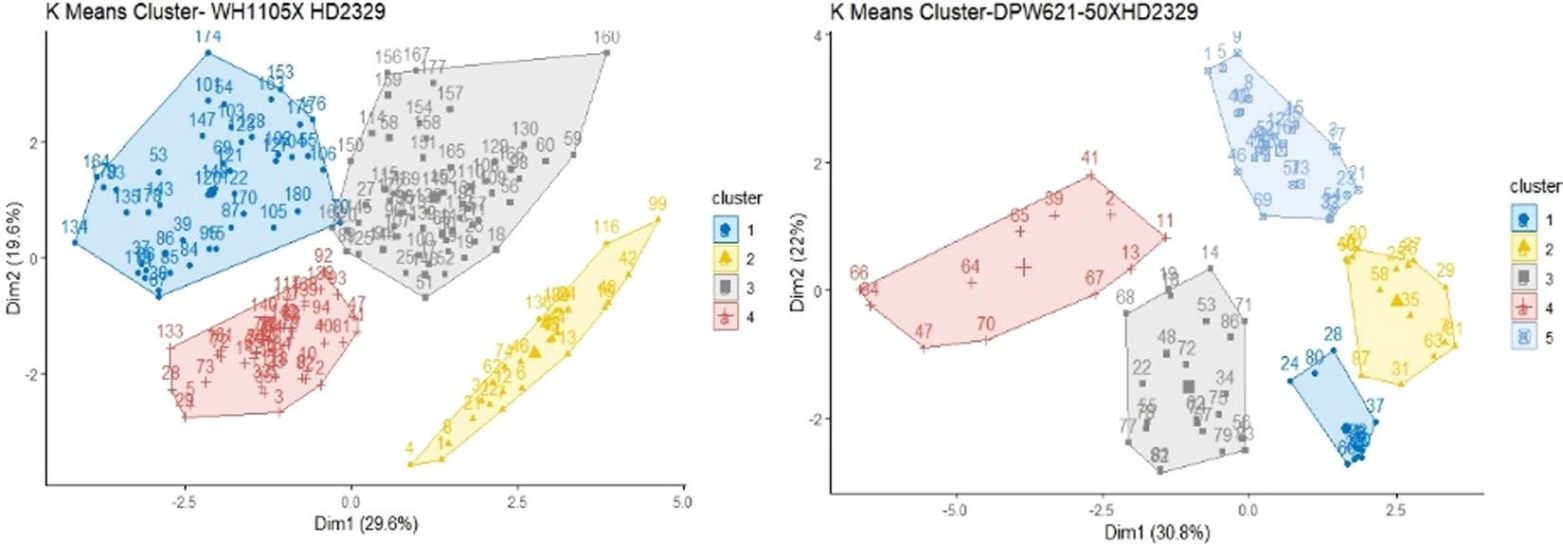
K- Means based clustering of populations on principal component axis.

The discriminant analysis of nine populations using K means and hierarchical clustering on two principal components population axis is given in (Fig. 4). The population WH1105 × HD2329 was classified into four groups with R^2^ = 50.0% explained by two principal components. The population HD3086 × HD2189 was classified into five clusters explaining about 50.5% of variance by two principal components. The third population HD2967 × RAJ4422 was classified into five clusters and the two principal components explained 59.2% variance.

The second line in Fig comprising populations DPW621–50 × HD2329, HD3086 × HD2987, and HD2932 × HUW666 was classified into five clusters explaining about 52% of variance by the two components.

The populations PBW373 × HD2329 was classified into four clusters and two components explained 48.3% variability. The other two combinations *viz*. PBW725 × PBW707 and K1301 × HD2851/Yr10 were classified into five clusters each explaining 54.4% and 55.6% variability by two components.

### Correlations

**Figure 5:**
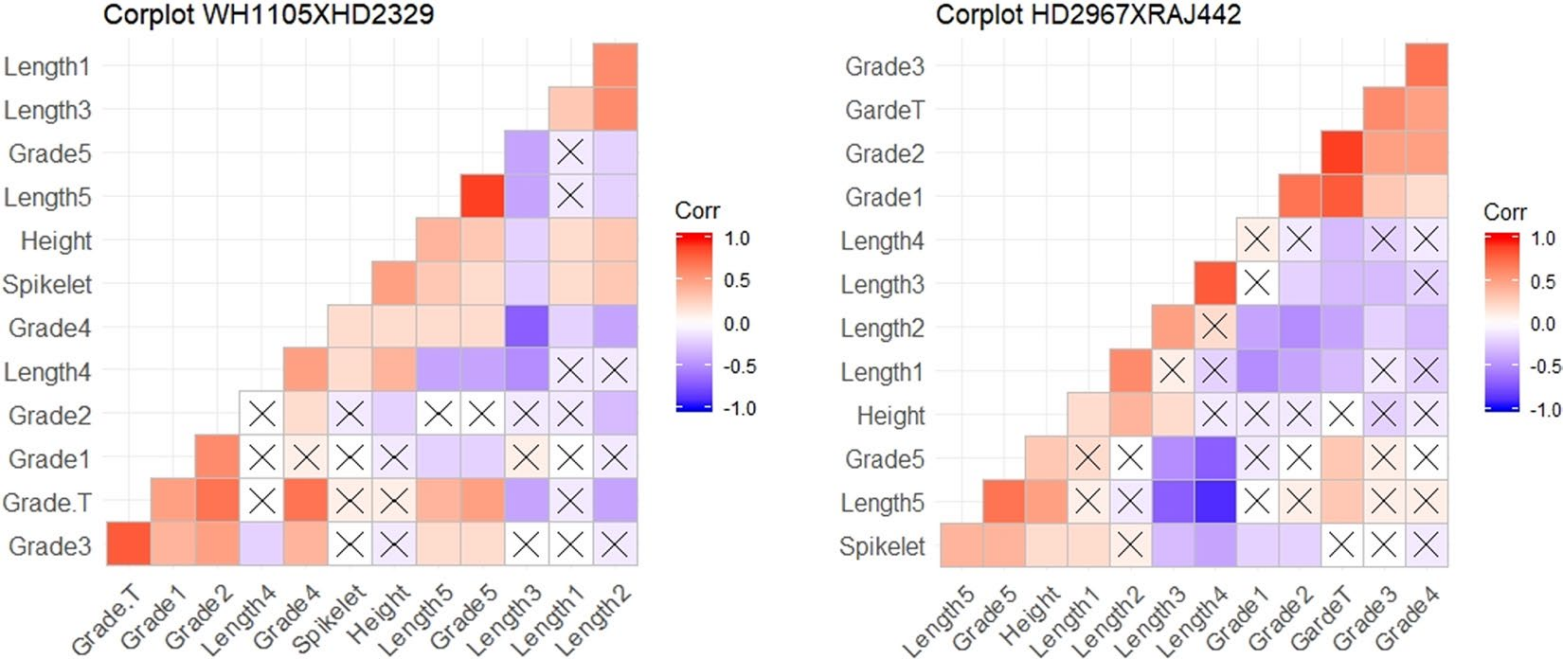
Hierarchical clustering and correlation among Internode lengths, height and solidness grades(*The respective numbers of length and grades refer to length and solidness grades starting from bottom internode and the Grade T refers to the total of the grade of all internodes of a given tiller).

The correlations among the solidness grades in each internode, internode length, spikelet number, length of tillers and total solidness grade were estimated and the same were classified following hierarchical clustering (Fig. 5). As the populations were segregating, measuring all the yield attributes was not feasible, therefore, only the spikelet number was considered as an indicator of sink capacity.

The spikelet number was found to be associated with the length of uppermost internode *i.e*. peduncle length and height in all the populations. Furthermore, the grade total representing the degree of solidness in tillers was not associated with spikelet number suggesting that solidness of tillers or internode is not negatively correlated to the sink capacity of the plant.

### Identification of desirable recombinants

**Figure 6:**
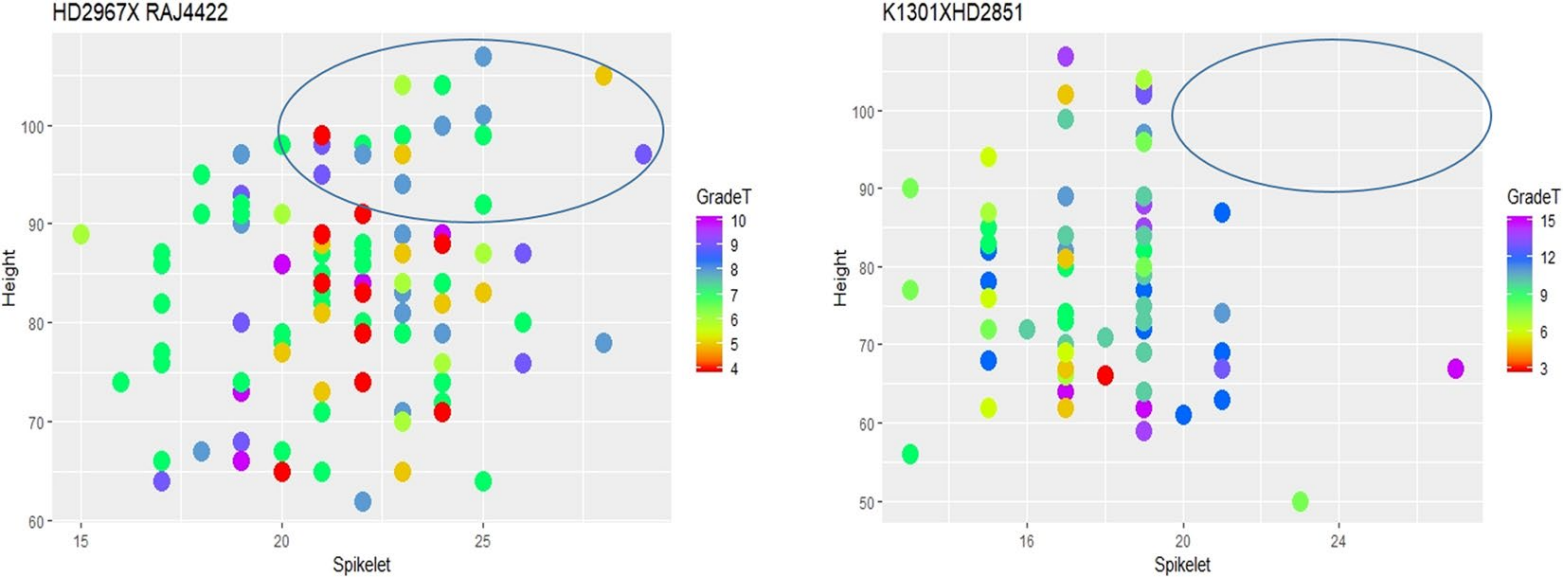
Graphical visualization for identification of desired recombinants (violet, blue and dark green) the individuals with solidness grade total of ≥8, spikelet number ≥20 and height ≥90 cm.

The number of desired recombinants varied in different populations indicating an array of combining ability for stem solidness with plant height and number of spikelets (Fig. 6). For instance, K1301 × HD2851 was the poorest combiner in which none of the genotypes with more than 90 cm were identified. All the crosses with HD2329 could reveal about 2% transgressive segregants with tillers having a high solid score with expression in at least three internodes, with more than 20 spikelets and height above 90 cm making the population skewed towards the parent HD2329 which has the height of 80–85 cm and average spikelet number of 15–18. The other populations falling in this category were PBW725 × PBW707 and HD2932 × HUW666. Therefore, increasing the size of the segregating population could help to identify more number of lines with desirable traits combination. The crosses HD3086 × HD2189, HD3086 × HD2987, and HD2967 × RAJ4442 were found to be better combiners with a higher number of useful recombinants.

## Discussion

Lesser number of solid stem genotypes among the very large number of F_1s_ explored for the traits indicate the lower frequency of favorable alleles for pithiness in the Indian breeding germplasm probably due to the non-integration of this trait in the selection criterion. It could also be due to the non-complementary distribution of favorable allele/transcription factors in the parental genotypes^21,22^.

The varieties HD2329, HD2733, and WH1175 were found to be better combiners for solid stem and therefore, can be further used as parents in the breeding program. The solid stem F_1s_ from hollow parents can not be explained by the theory of dominance as indicated in many of the previous studies Most of these studies were based on the behavior of a single cross involving solid and hollow genotypes^9,10,23,24^.

The pithiness expressivity is variable within a tiller as well as among the tillers of a single plant in F_1_ combinations. This can be explained by the complementation of additive factors dispersed among the two parents and a strong influence of transcription factors by the environment. The increased copy number of the *TraesCS3B01G608800* (DNA-binding one-zinc finger) transcription factor has been found associated with solid-stem phenotypic variation^25^. In addition to the genetic components, the stem solidness is subject to variable expression due to different external non-genetic factors, such as seeding rate and N- application^26–28^. There is also a possibility of different allelic interactions of main factors as well as transcription factors or the main effect QTL on 3BL and minor QTLs including Qss.msub-3DL^29,30^. However, with the available information on the genetic loci and markers linked with solid stem expression, it is possible to select parents for making crosses more precisely^8,9,25^.

The best-fit regression equation has been used to express the relative change in solidness^26^. The solidness is expressed based on the genomic constitution of wheat lines as different genomes interact to give a different degree of expression of solidness^24,30^. The haplotype-based comparison indicated a clear binomial distribution in DH lines from Durum solid × hollow combination while in case of common wheat the distribution was near binomial with more tendency towards being hollow to semisolid^9^. It may be due to some transcription factors/modifiers which modify the expression of pith production based on certain non-genetic factors including gravity. It is highly likely that stem solidness might interact and maintain the trade-off with other plant architectural traits, such as stem elongation and tillering capacity.

The K-means based clustering, which utilizes the Euclidean distance and centroid information to classify the individuals into different groups, is considered as a powerful tool to visualize and compare the underlying differences among individuals. These groups are graphically depicted on two principal components axes that explain the maximum variability. This method is also known as discriminant analysis of principal component (DAPC)^31^. The DAPC or PCA based clustering is a strong tool being used in genetic studies based on both genotypic and phenotypic data^31–33^. The method gives an idea of possible phenotypic categories in a population of the variables used in the study. In this study, the nine populations could be optimally classified in four to five clusters indicating the role of at least two or more quantitative factors with a variable degree of contribution for solidness. This can be substantiated by the theory of two QTLs, one being major and other being minor governing the major portion of solidness^16,29^. The remaining part of variance can be attributed to the other possible minor QTLs and interaction effects of QTL × genotypic background^9^.

The present study is based on different genetic combinations and could find almost all kinds of associations reported in the past. In the segregating population of the cross K1301 × HD2851, segregants were either with low spikelets number coupled with solidness or reduced height with increased spikelets number and solidness, again putting a ceiling for yield gain due to lower sink in one case and lower source in another case. Similarly, in the case of WH1105 × HD2329, segregants with a significant reduction in height with increased spikelets and solidness were found. The significant negative association between solid stem and yield were reported by many workers in the past^13,14,34^. Studies with solid-stem × hollow-stem wheat crosses have also shown that stem solidness tends to be negatively correlated with plant height^35^. However, in the segregating population of HD2967 × RAJ 4422 the frequency of favorable segregants with optimum plant height, increased spikelet number, and more stem solidness were quite high. Nevertheless, it is imperative to say that the recovery of useful recombinants is determined not only by the segregating population size but also it depends upon the biological tradeoffs among these traits. Favorable recombinant in this population indicates the possibility of yield gain with decreased incidence of stem lodging due to stem solidness and is supported by other workers^21,35^ also. Similar results have also been reported in solid stem cultivars developed at the Montana Agricultural Experimental Station, the USA is an example of this^17–20^. Interestingly, the landmark green revolution varieties in India, Kalyan Sona and HD2329 were also solid stemmed. Therefore, the genotypes with high yield potential can be derived from crosses having favorable solidness and sink size with supporting height and biomass.

## Conclusion

Lodging has become one the biggest stumbling block in minimizing the gap between realized and potential yield in the high yielding environment of NPWZ of India. The solid stem was never been explored by Indian breeders because of no major threat from stem sawfly, borer insects and comparatively lower incidence of lodging. The understanding of the negative correlation between stem solidness and grain yield also played a role in keeping the breeding aim at bay. However, the role of stem solidness towards improving plant strength to support lodging tolerance and providing better mobilization of stem reserves under stress conditions besides sawfly resistance have been advocated in the recent past. The current study is a proof of concept that the solid stem genotypes can be developed from a large number of hollow but improved genotypes by combining the dispersed favorable alleles for stem solidness in different genetic backgrounds. Our results indicate that increased phenotypic expression of stem solidness was large because of quantitative factors with additive nature rather than dominance or recessive kind of gene action. The occurrence of fewer transgressive segregants for improved sink and stem solidness in some of the crosses indicates the possibilities of their selection by increasing the size of segregating populations. A better understanding of the molecular basis of variable traits expression in the current segregating population of this study and newer targeted cross by integrating already available information on the role of transcription factors and genomic regions can speed up the yield gain by combining improved sink with lodging resistance.

## Supplementary information

Supplementary Dataset 1.

Supplementary Dataset 2.
