# Supplementary material for: Additive Genetic Behavior of Stem Solidness in Wheat (Triticum aestivum L.)
Source: Sci Rep. 2020 Apr 30;10:7336. doi: 10.1038/s41598-020-64470-x (PMC7193624; doi:10.1038/s41598-020-64470-x)
Supplement: Supplementary file 2 — Supplementary Dataset 2. [file 41598_2020_64470_MOESM2_ESM.pdf]

Additive Genetic Behavior of Stem Solidness in Wheat (*Triticum aestivum* L.)

Naresh Kumar Bainsla[1]*, Rajbir Yadav[1], G. P. Singh[1,2], Ram Kumar Sharma[1]

[1]Division of Genetics, ICAR- Indian Agricultural Research Institute, New Delhi-110012
[2]Indian Institute of Wheat and Barley Research, Karnal-132001

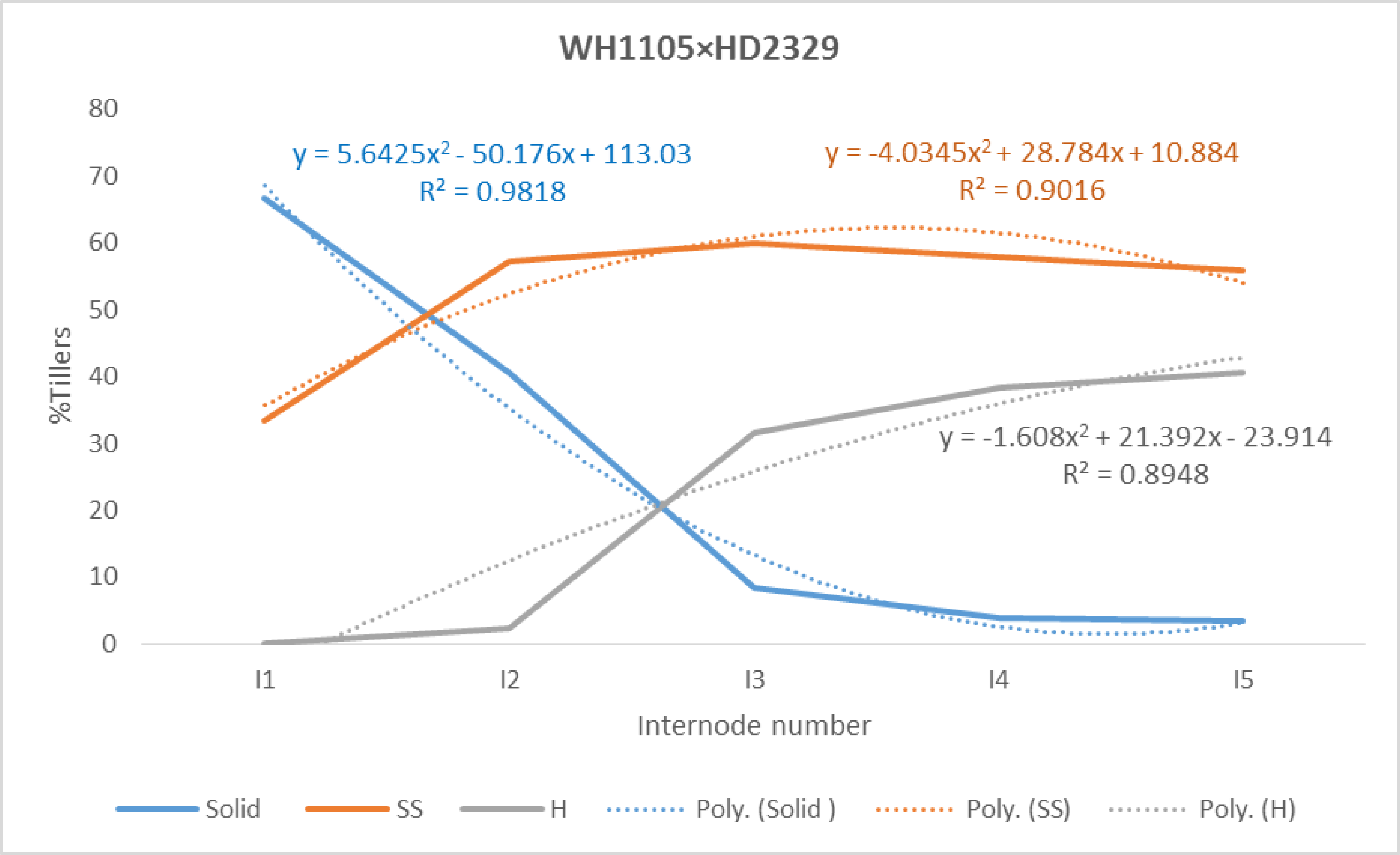

Figure 1.1 WH1105× HD2329

(The Y-axis shows the % tillers expressing the grade of solidness and X-axis shows the internodes starting from bottom to top. The dotted line shows the regression line while the solid line shows the data trend)

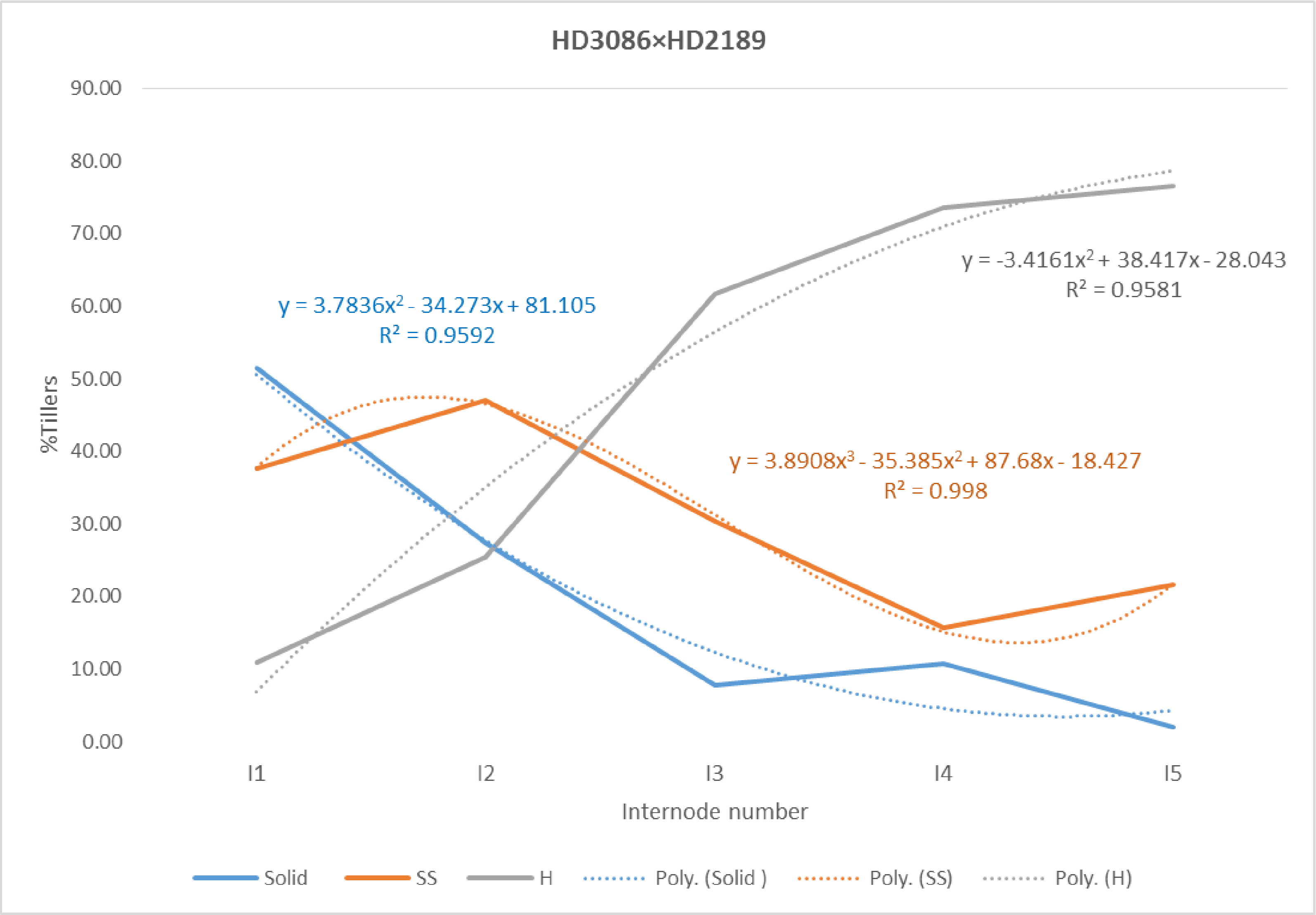

Figure 1.2 HD3086 × HD2189

(The Y-axis shows the % tillers expressing the grade of solidness and X-axis shows the internodes starting from bottom to top. The dotted line shows the regression line while the solid line shows the data trend)

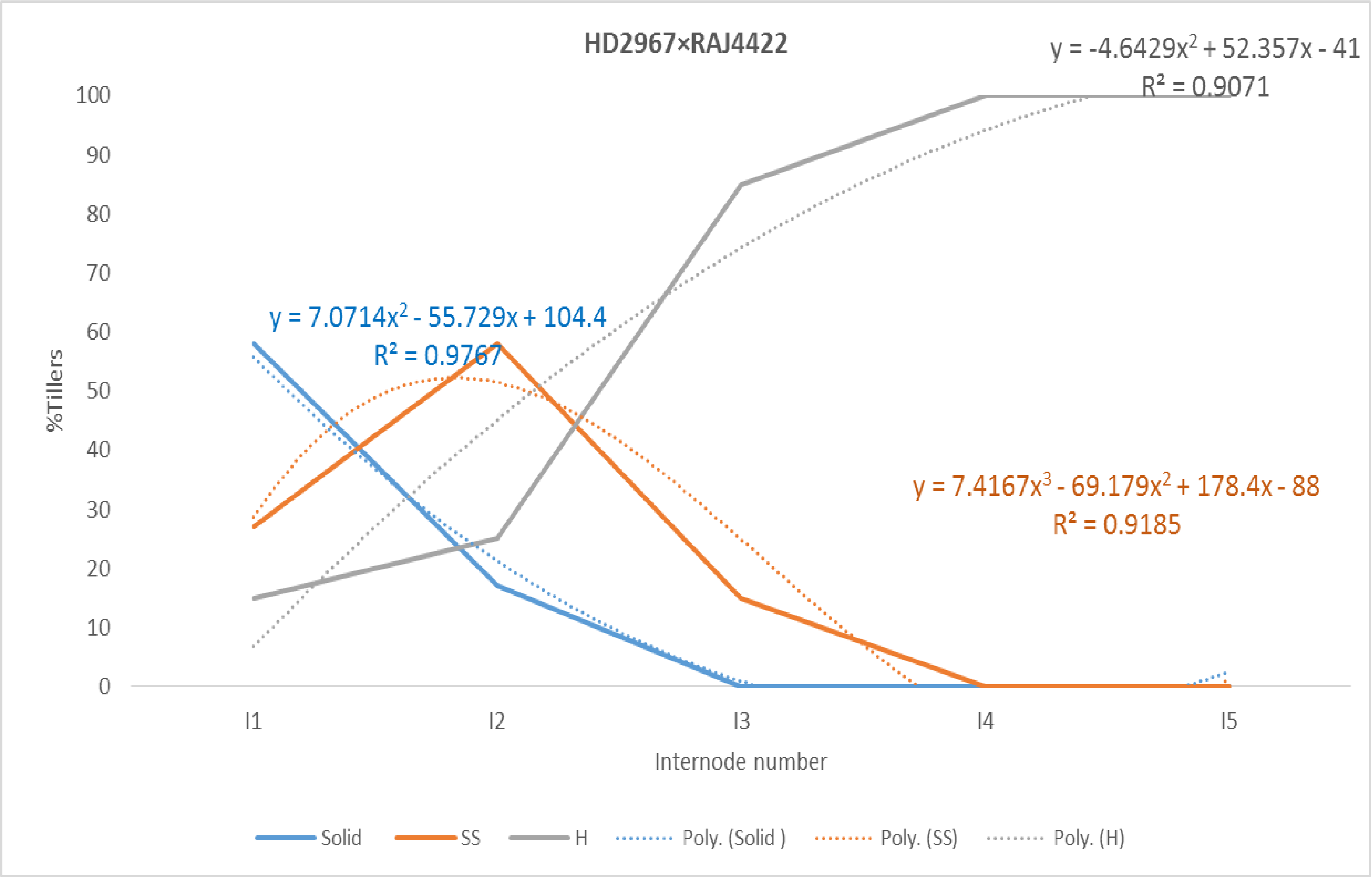

Figure 1.3 HD2967 × RAJ4422

(The Y-axis shows the % tillers expressing the grade of solidness and X-axis shows the internodes starting from bottom to top. The dotted line shows the regression line while the solid line shows the data trend)

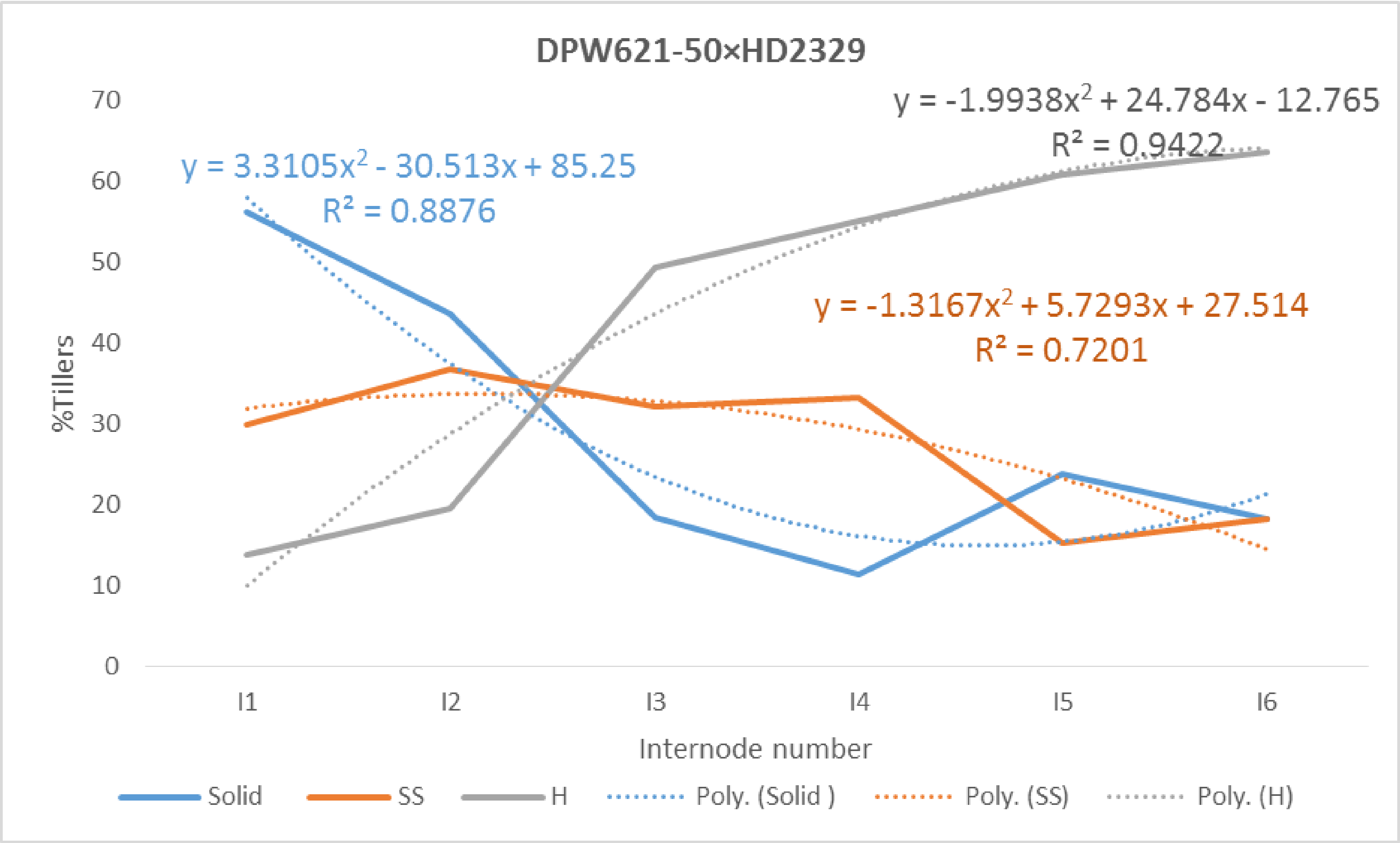

Figure 1.4 DPW621-50 × HD2189

(The Y-axis shows the % tillers expressing the grade of solidness and X-axis shows the internodes starting from bottom to top. The dotted line shows the regression line while the solid line shows the data trend)

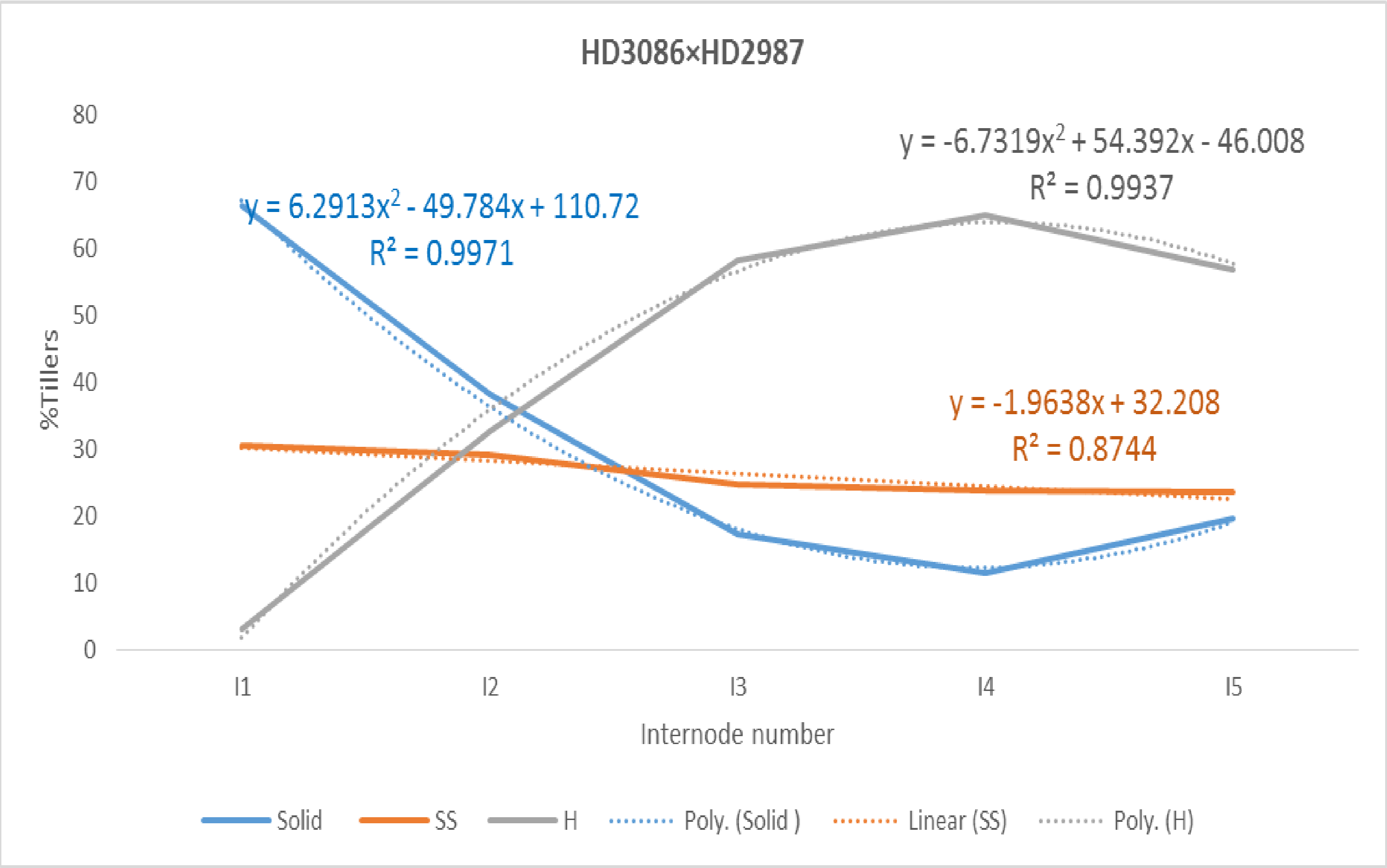

Figure 1.5 HD3086 × HD2987

(The Y-axis shows the % tillers expressing the grade of solidness and X-axis shows the internodes starting from bottom to top. The dotted line shows the regression line while the solid line shows the data trend)

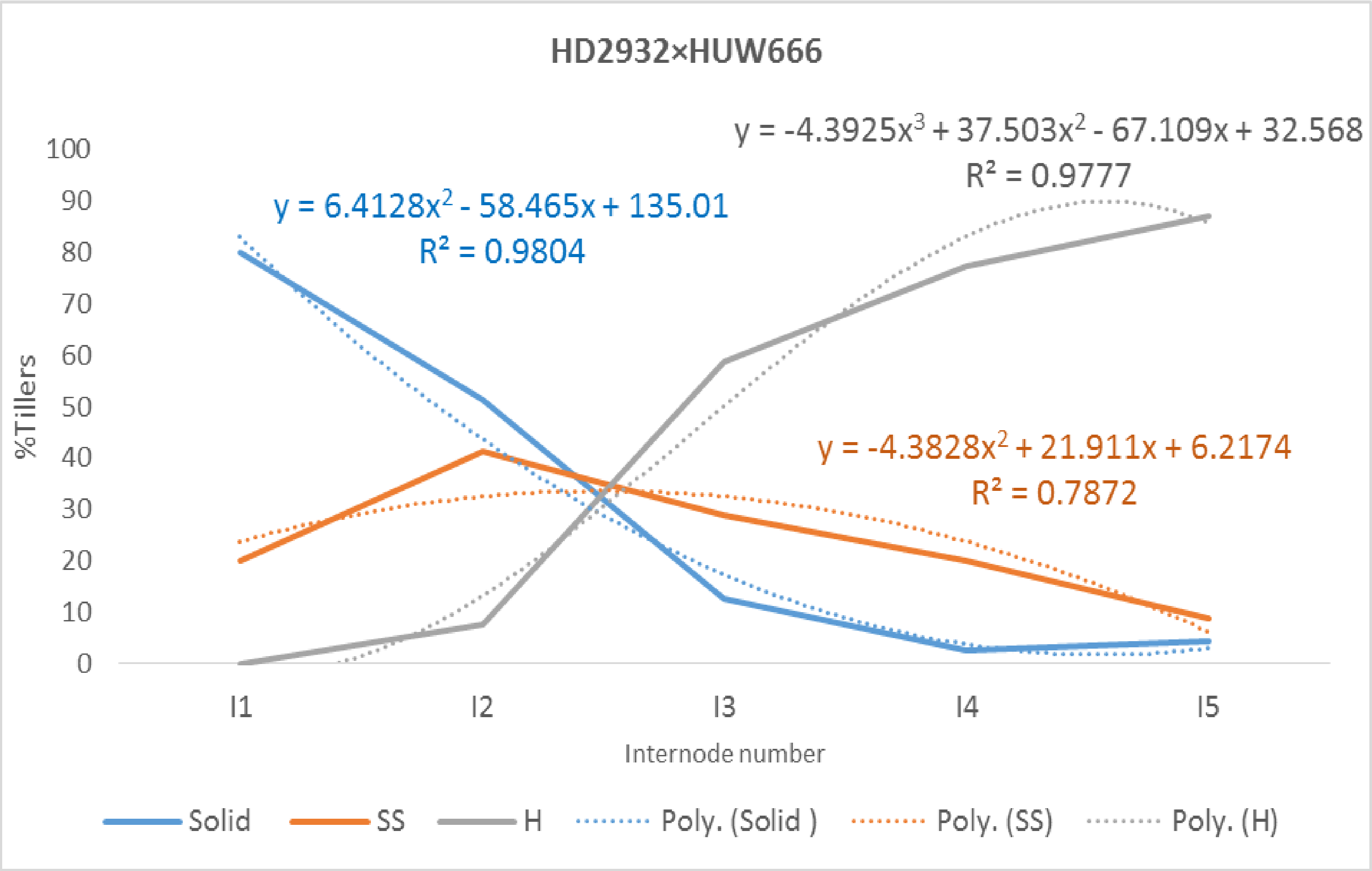

Figure 1.6 HD2932 × HUW666

(The Y-axis shows the % tillers expressing the grade of solidness and X-axis shows the internodes starting from bottom to top. The dotted line shows the regression line while the solid line shows the data trend)

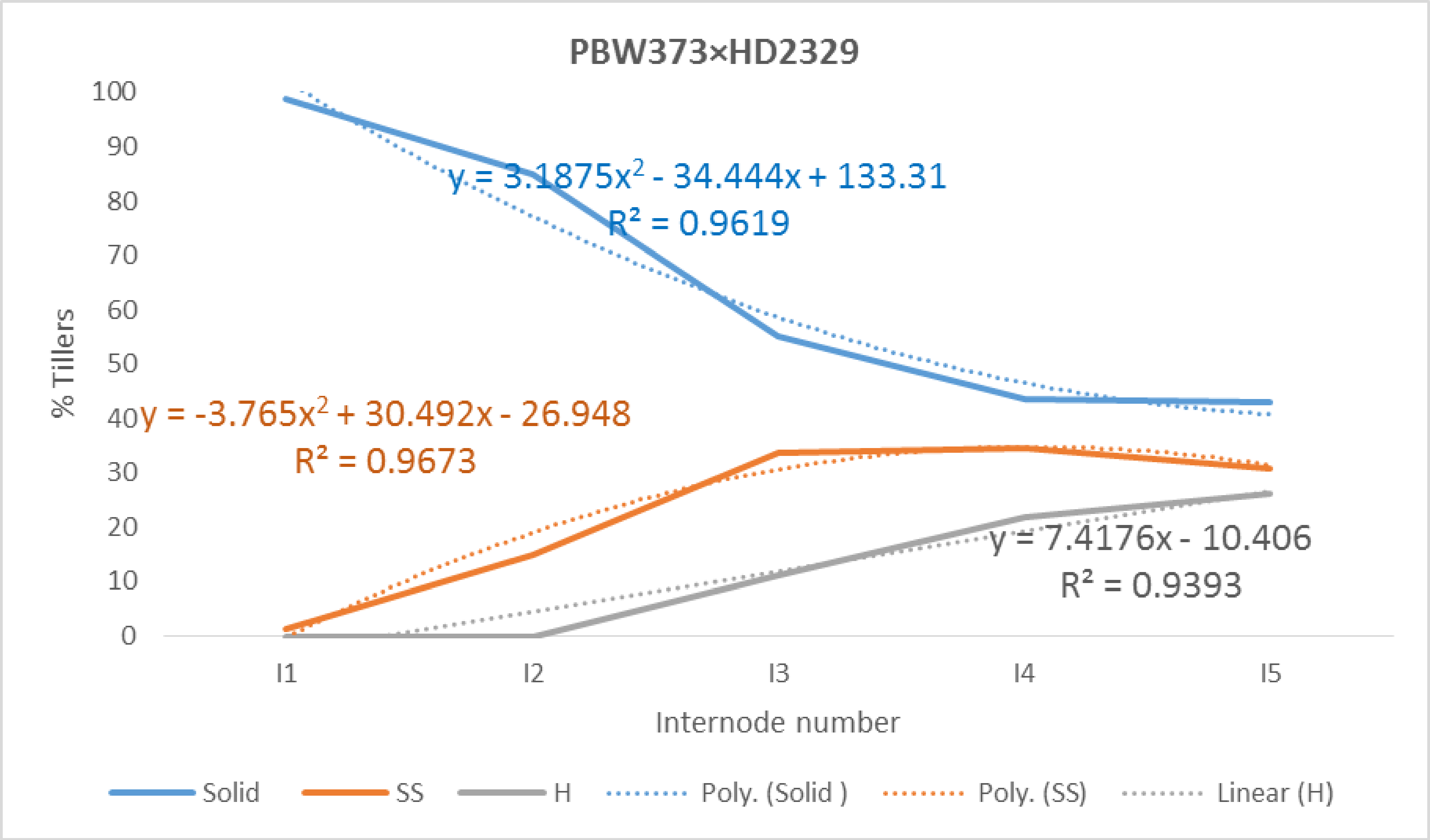

Figure 1.7 PBW373 × HD2329

(The Y-axis shows the % tillers expressing the grade of solidness and X-axis shows the internodes starting from bottom to top. The dotted line shows the regression line while the solid line shows the data trend)

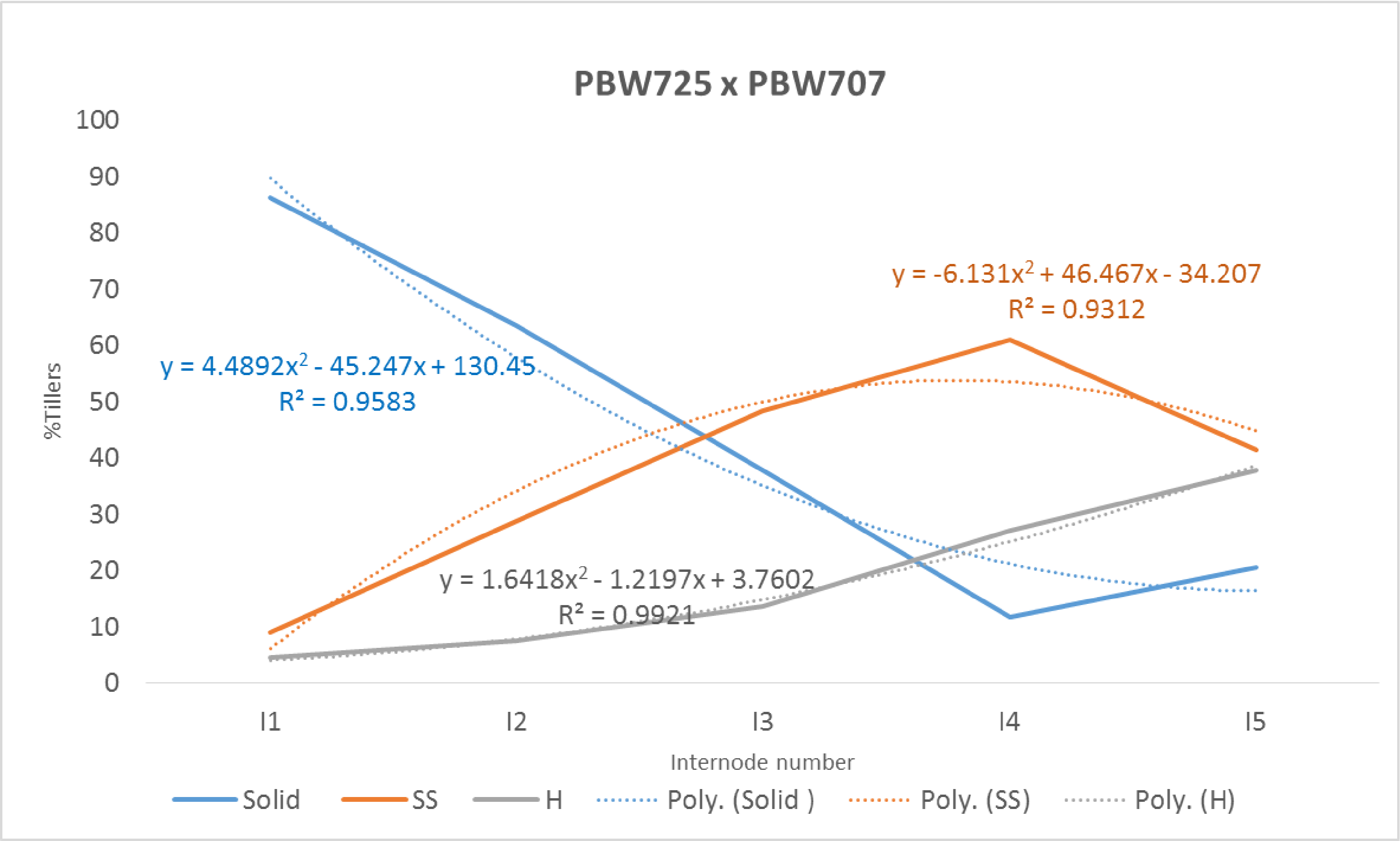

Figure 1.8 PBW725 × PBW707

(The Y-axis shows the % tillers expressing the grade of solidness and X-axis shows the internodes starting from bottom to top. The dotted line shows the regression line while the solid line shows the data trend)

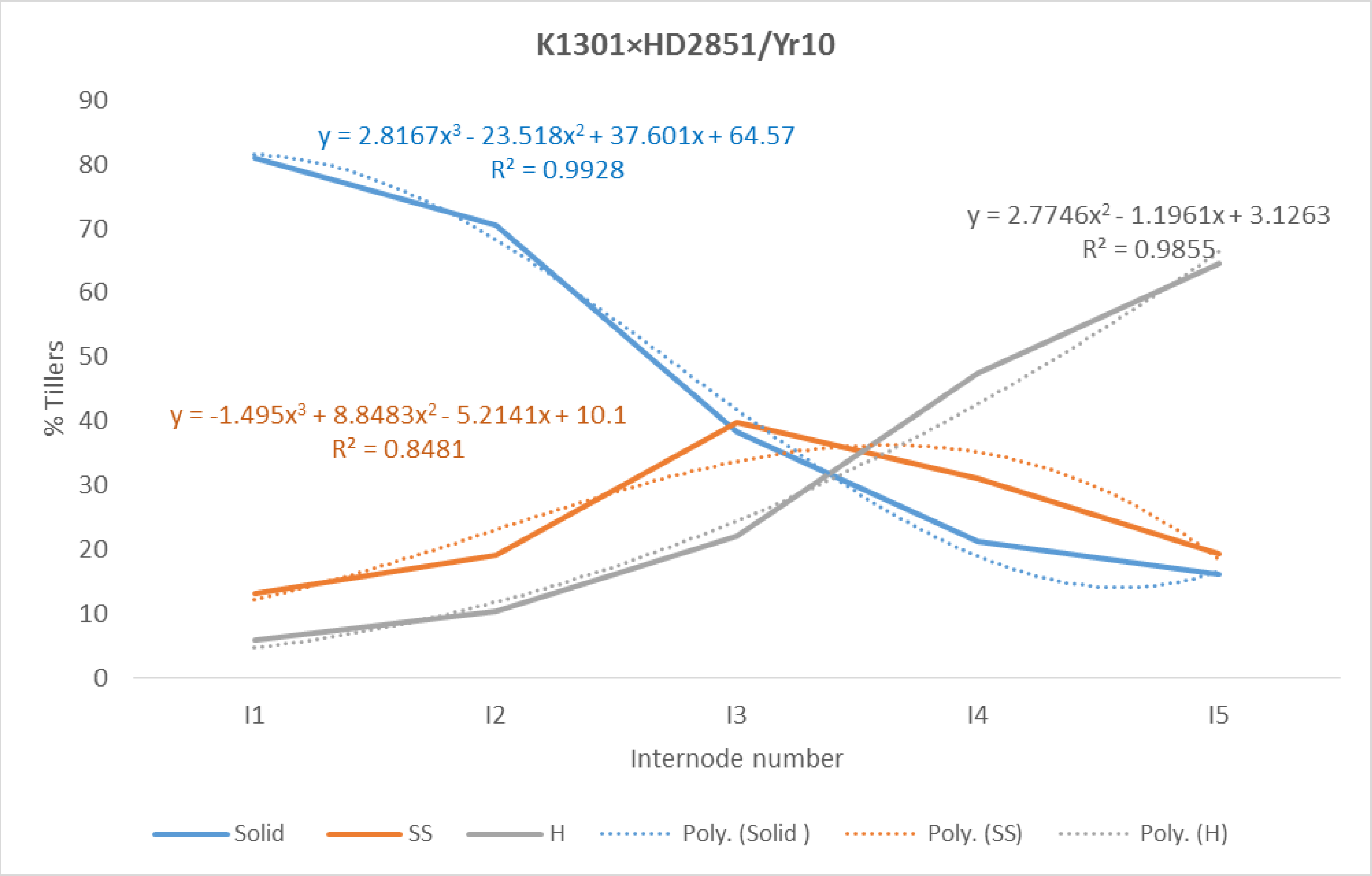

Figure 1.9 K1301 × HD2851/Yr10

(The Y-axis shows the % tillers expressing the grade of solidness and X-axis shows the internodes starting from bottom to top. The dotted line shows the regression line while the solid line shows the data trend)

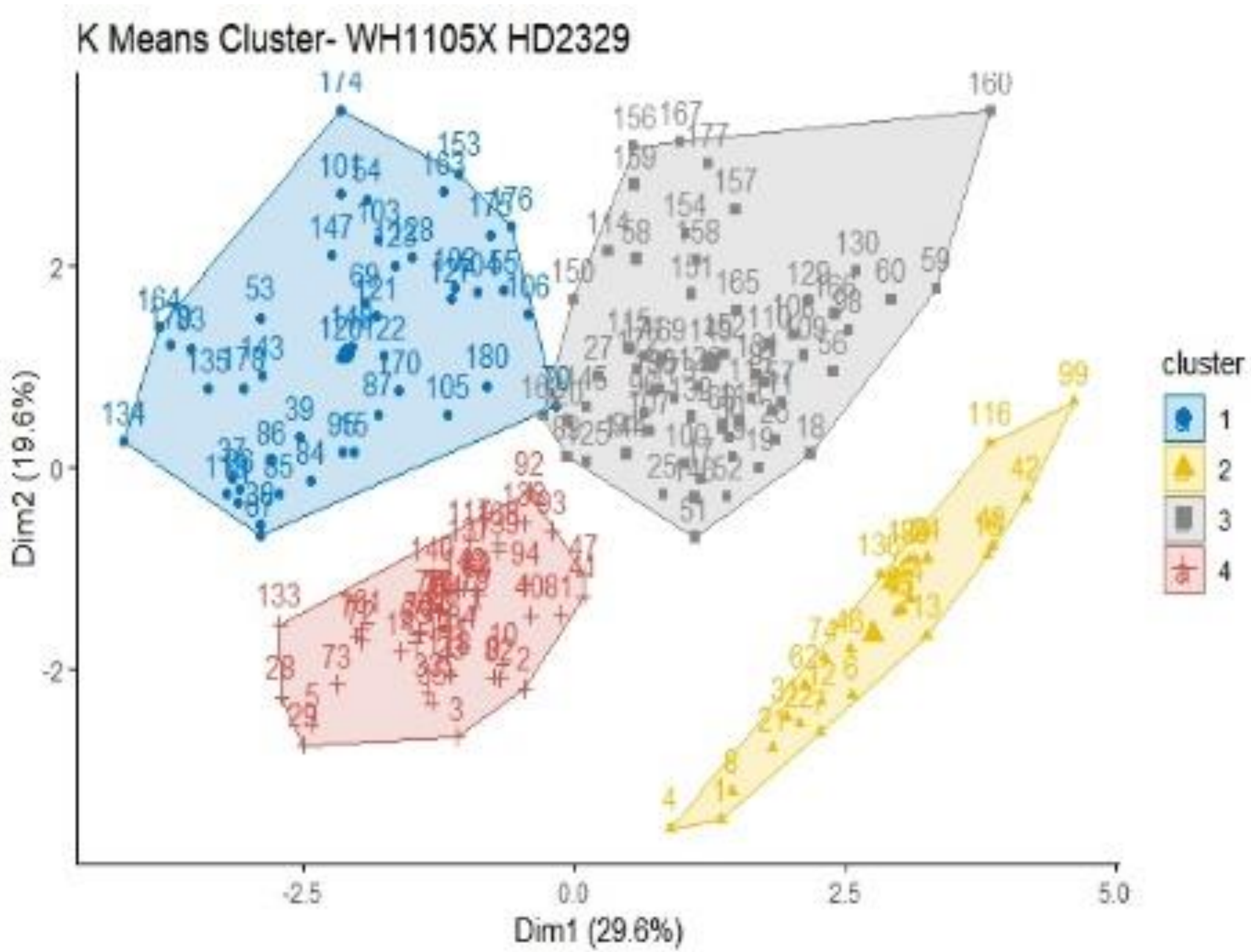

Figure 4.1 WH1105× HD2329
K- Means based clustering of population on principal component axis

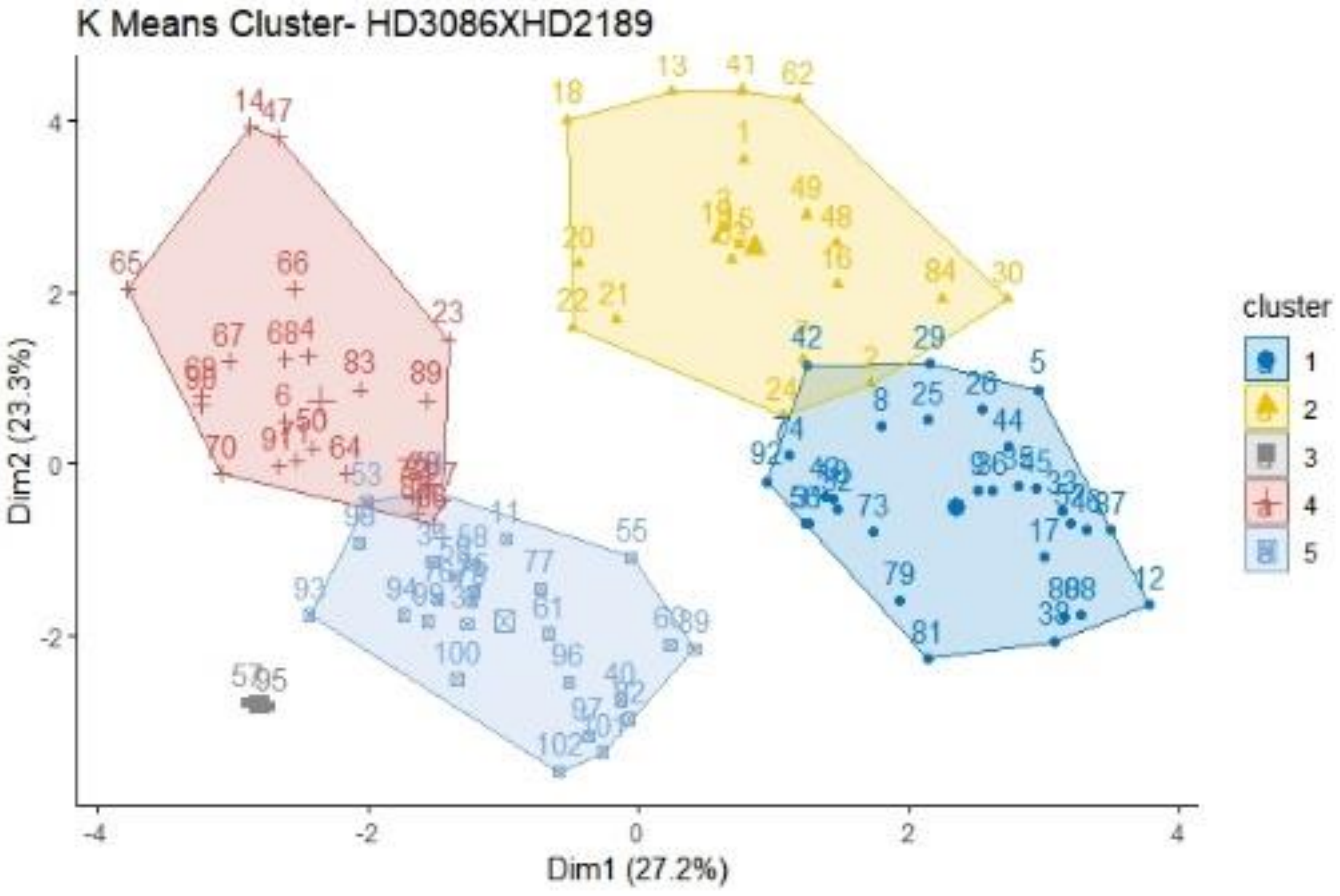

Figure 4.2 HD3086 × HD2189
K- Means based clustering of population on principal component axis

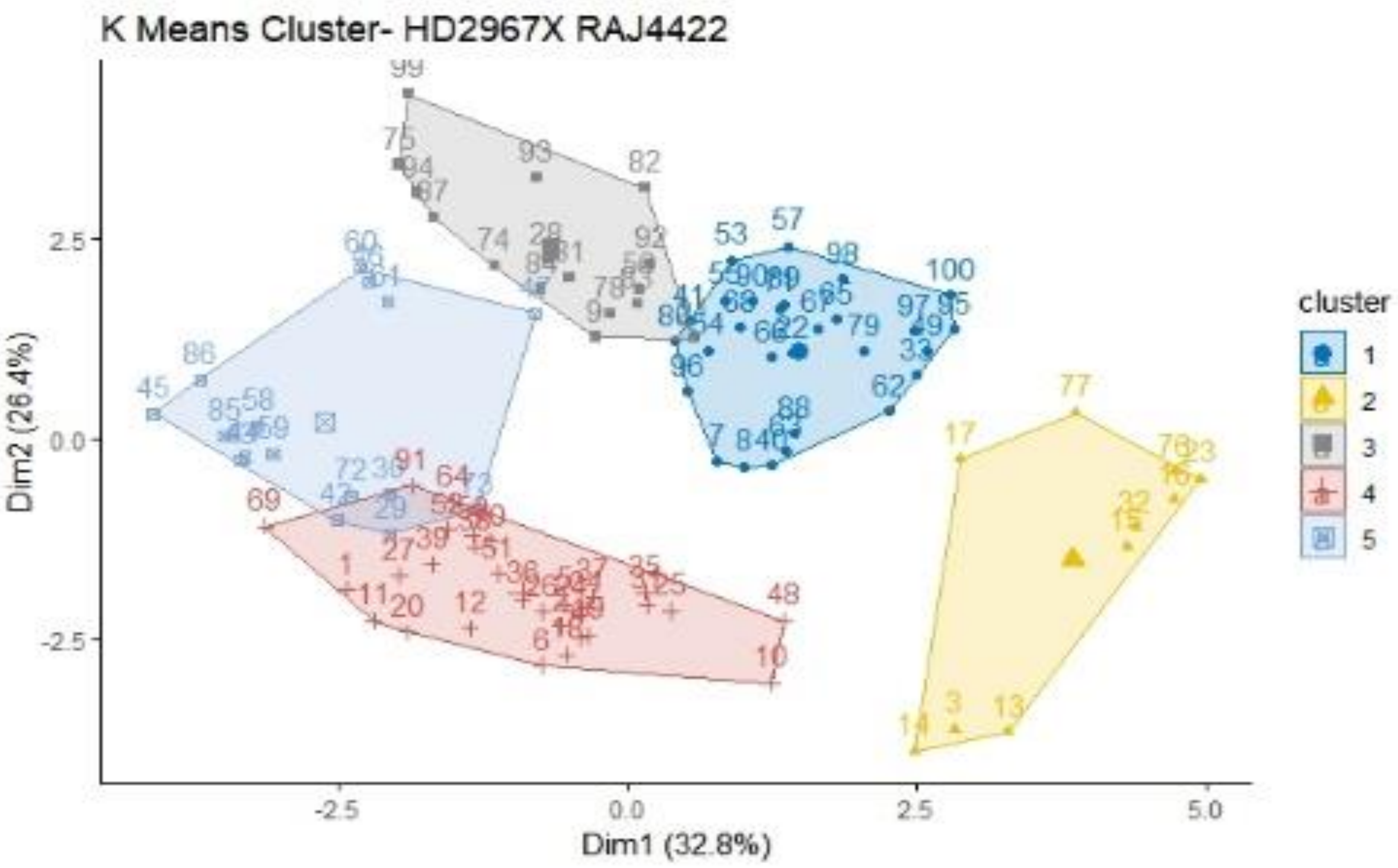

Figure 4.3 HD2967 × RAJ4422
K- Means based clustering of population on principal component axis

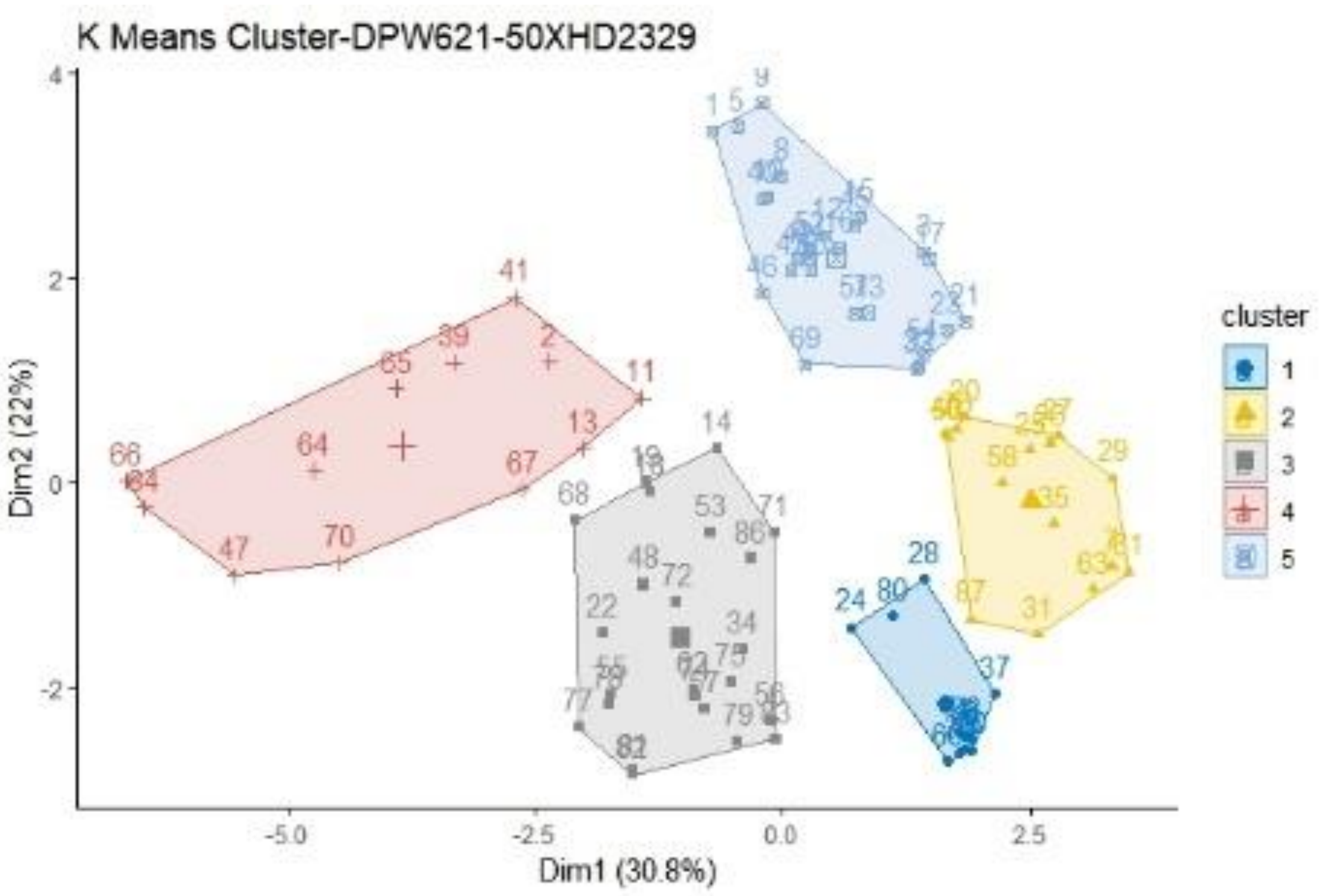

Figure 1.4 DPW621-50 × HD2189
K- Means based clustering of population on principal component axis

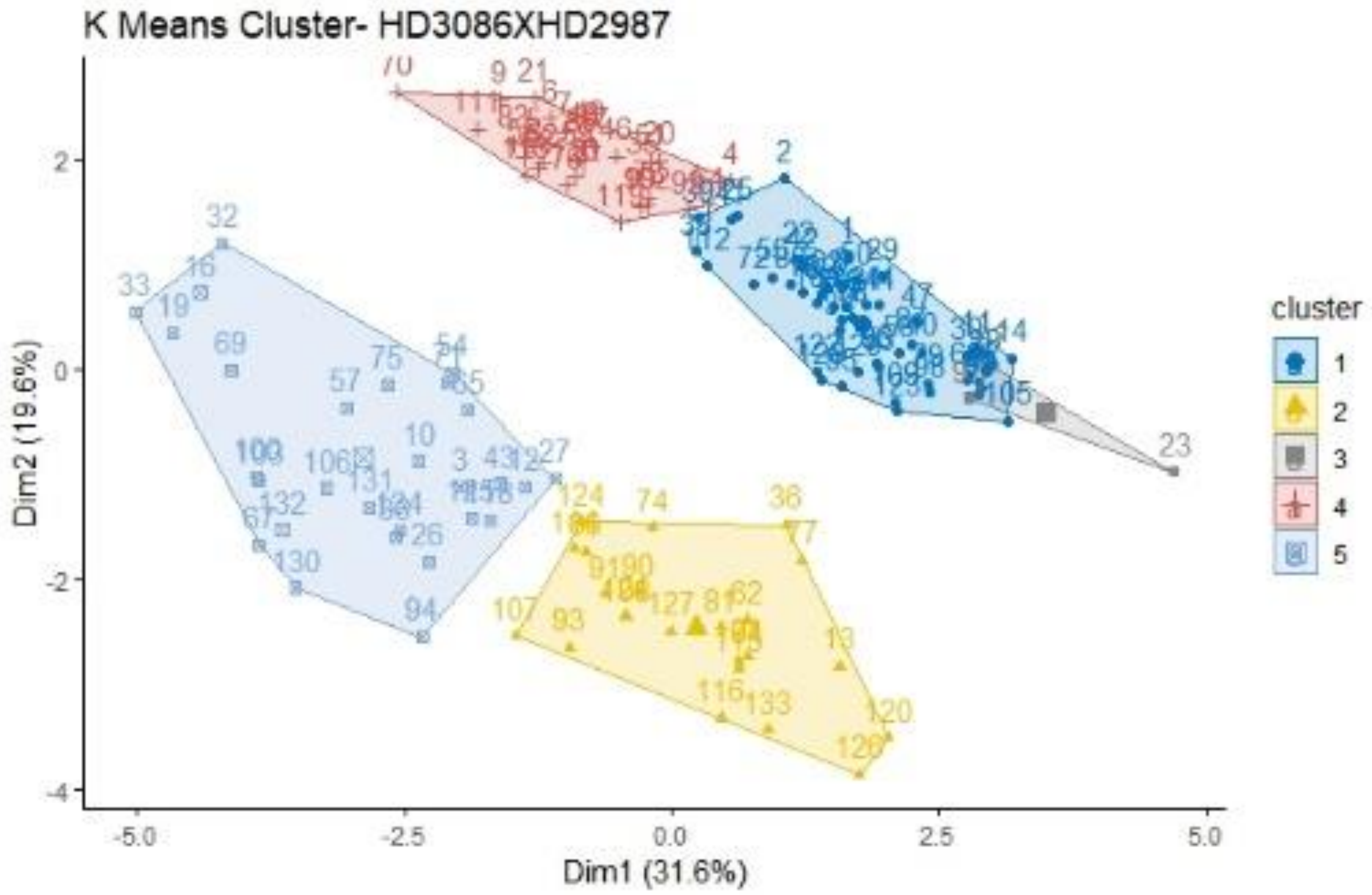

Figure 4.5 HD3086 × HD2987
K- Means based clustering of population on principal component axis

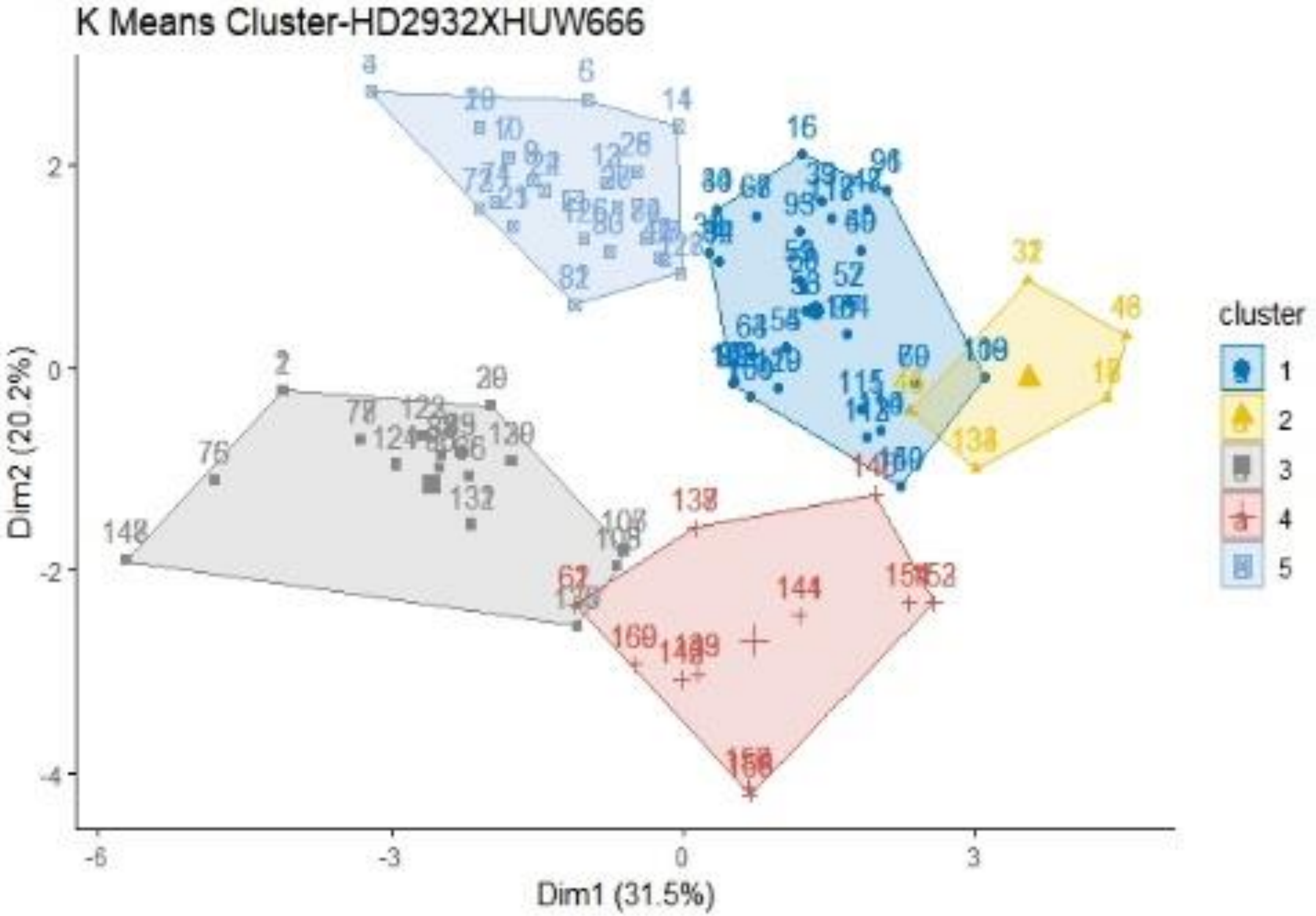

Figure 4.6 HD2932 × HUW666
K- Means based clustering of population on principal component axis

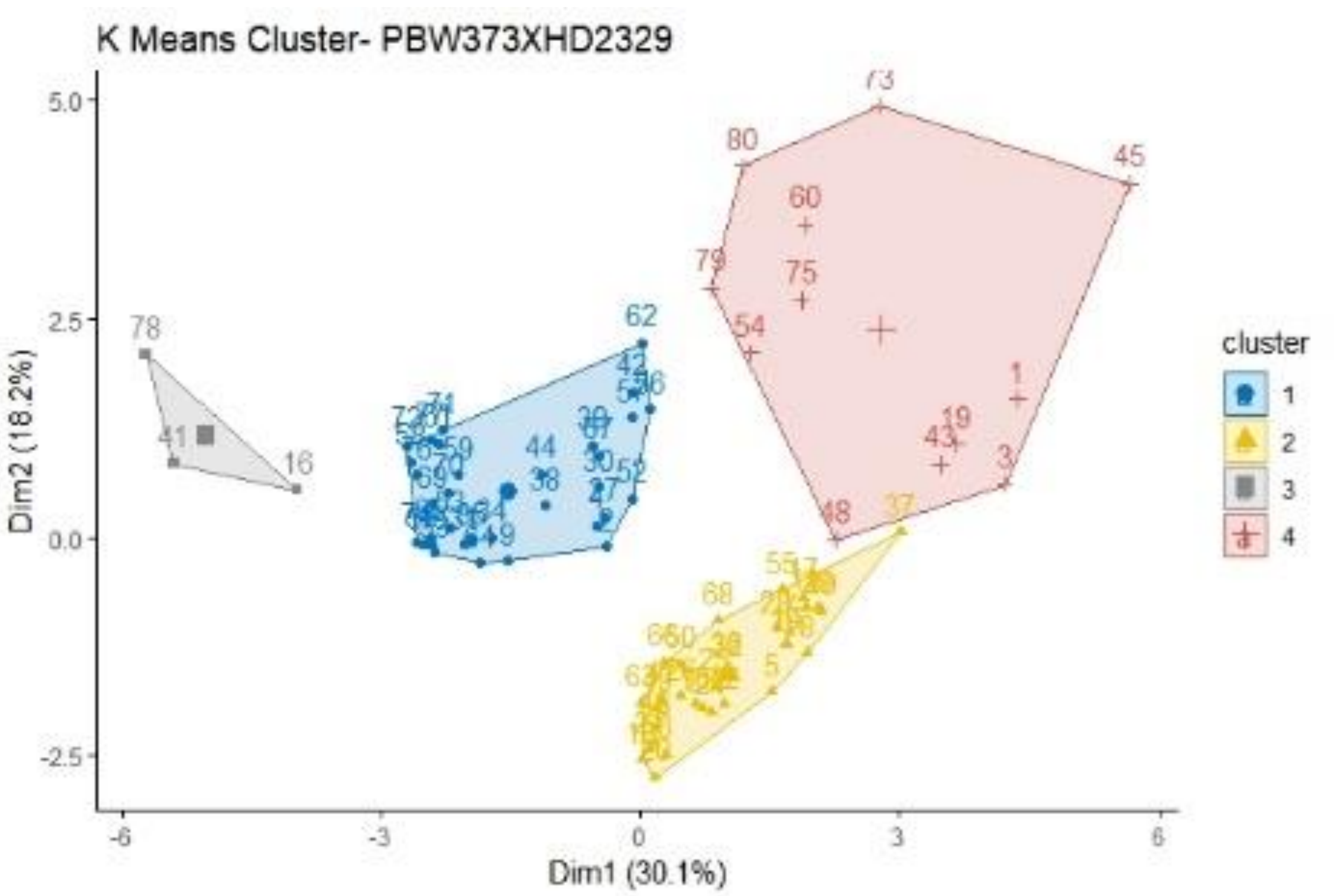

Figure 4.7 PBW373 × HD2329
K- Means based clustering of population on principal component axis

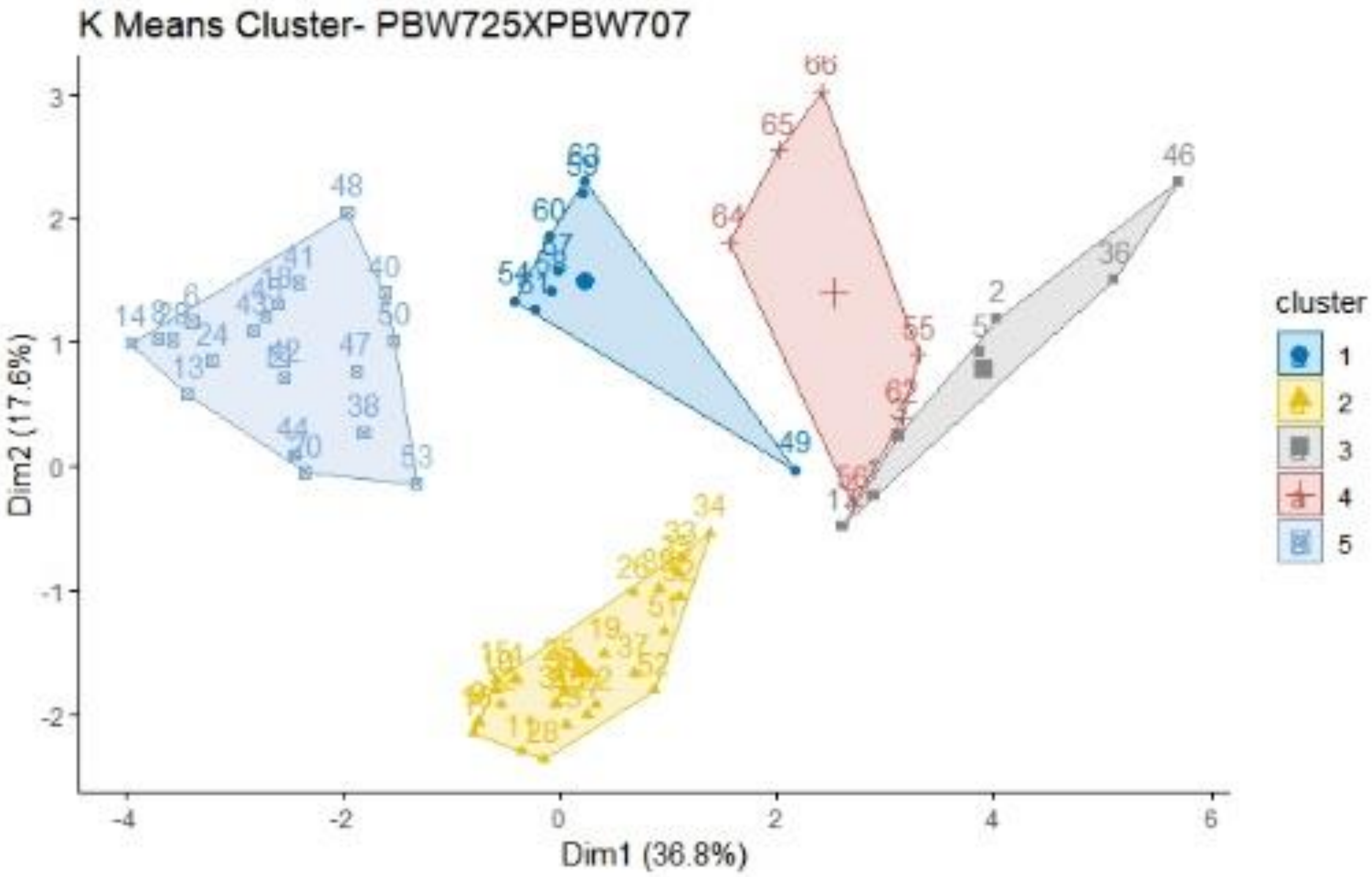

Figure 4.8 PBW725 × PBW707
K- Means based clustering of population on principal component axis

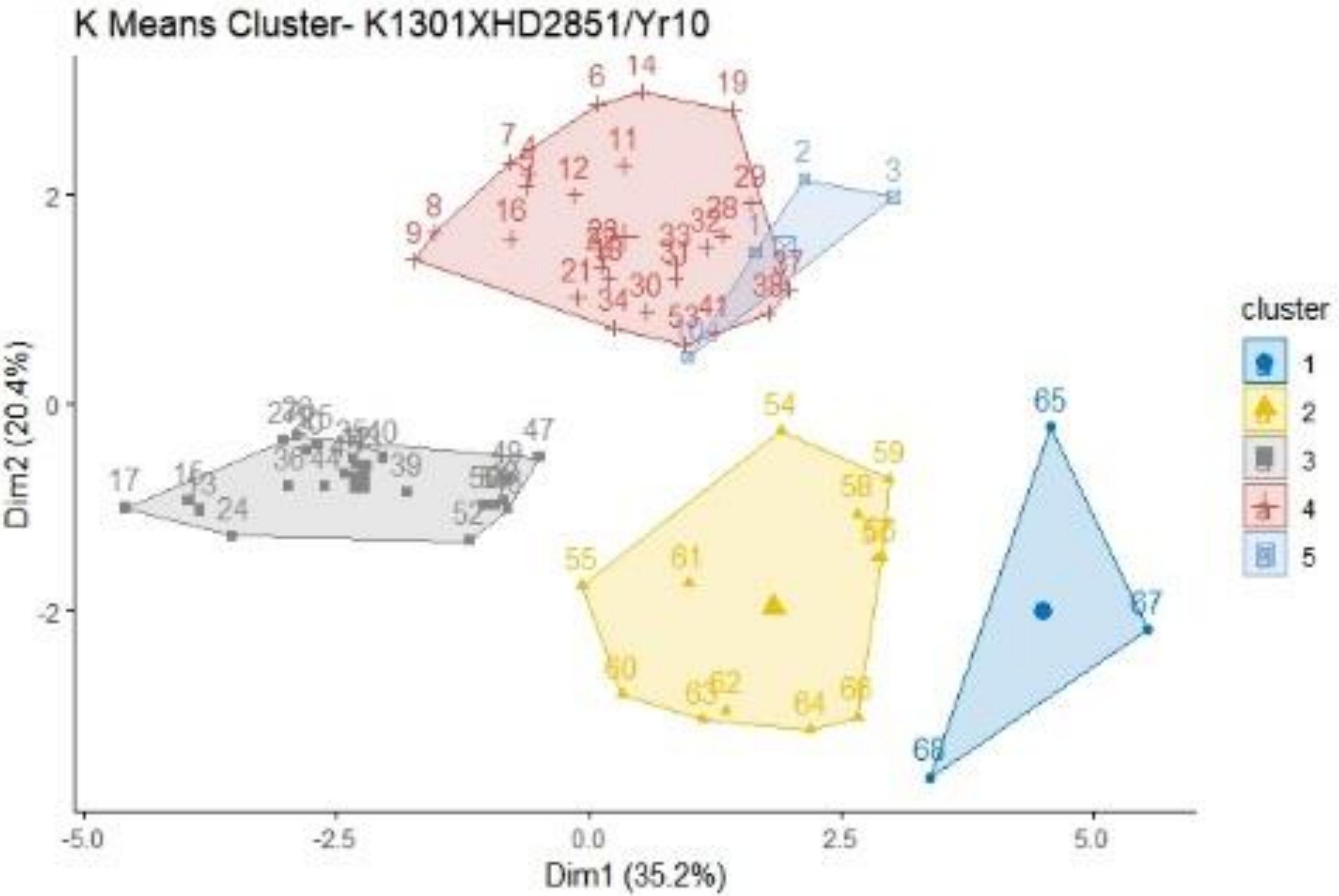

Figure 4.9 K1301 × HD2851/Yr10
K- Means based clustering of population on principal component axis

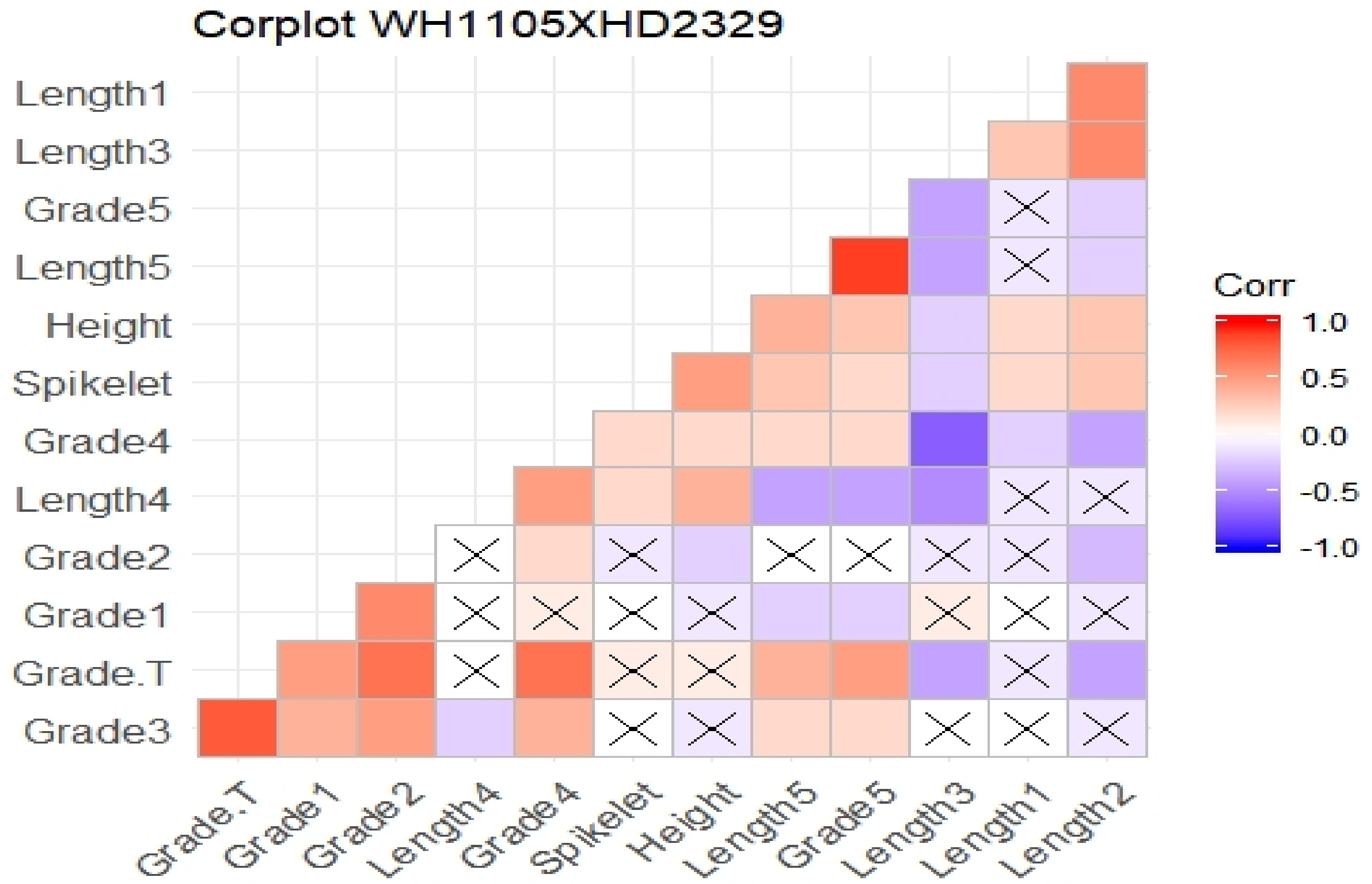

Figure 5.1 WH1105× HD2329

Hierarchical clustering and correlation among Internode lengths, height and solidness grades

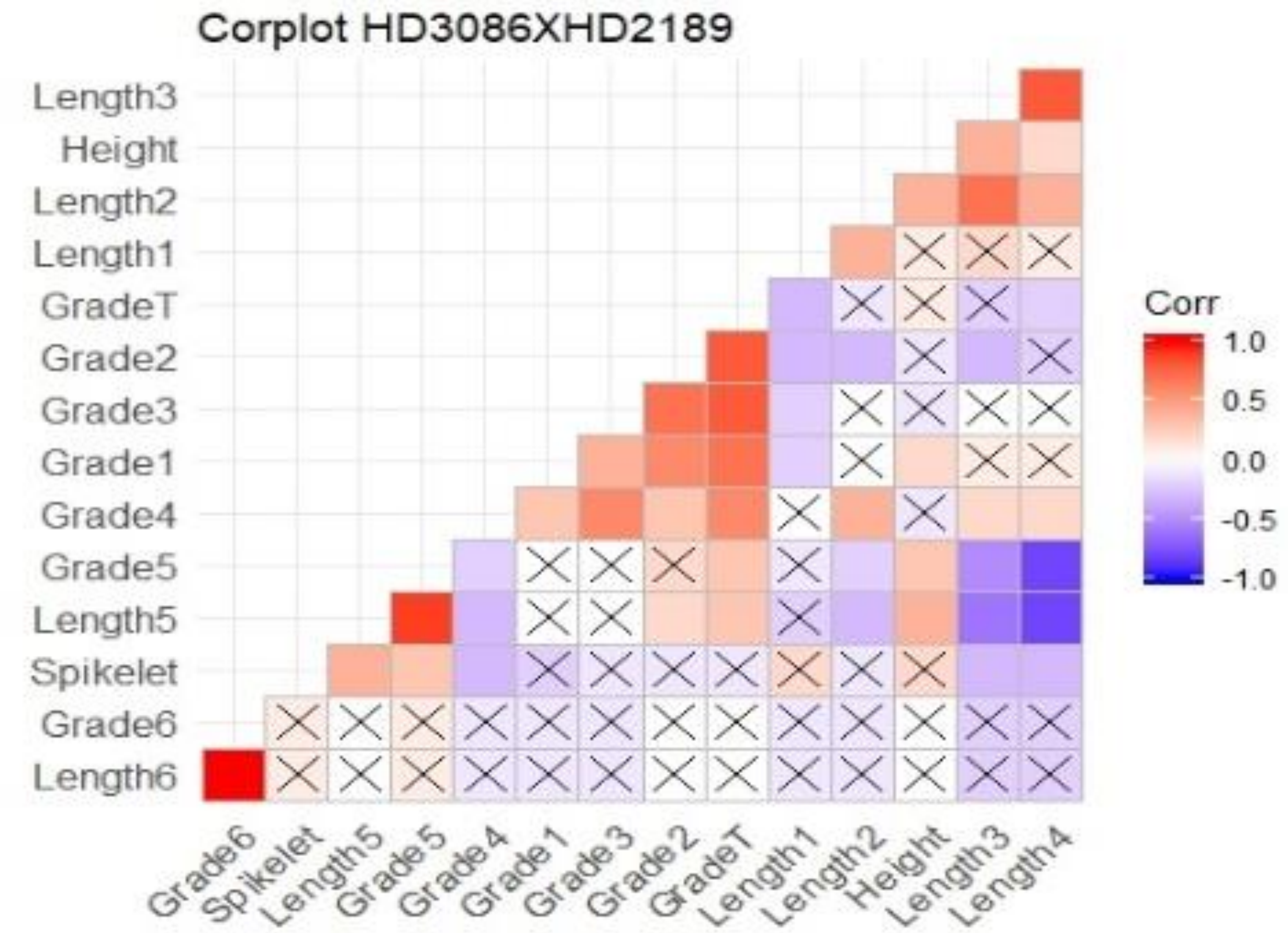

Figure 5.2 HD3086 × HD2189
Hierarchical clustering and correlation among Internode lengths, height and solidness grades

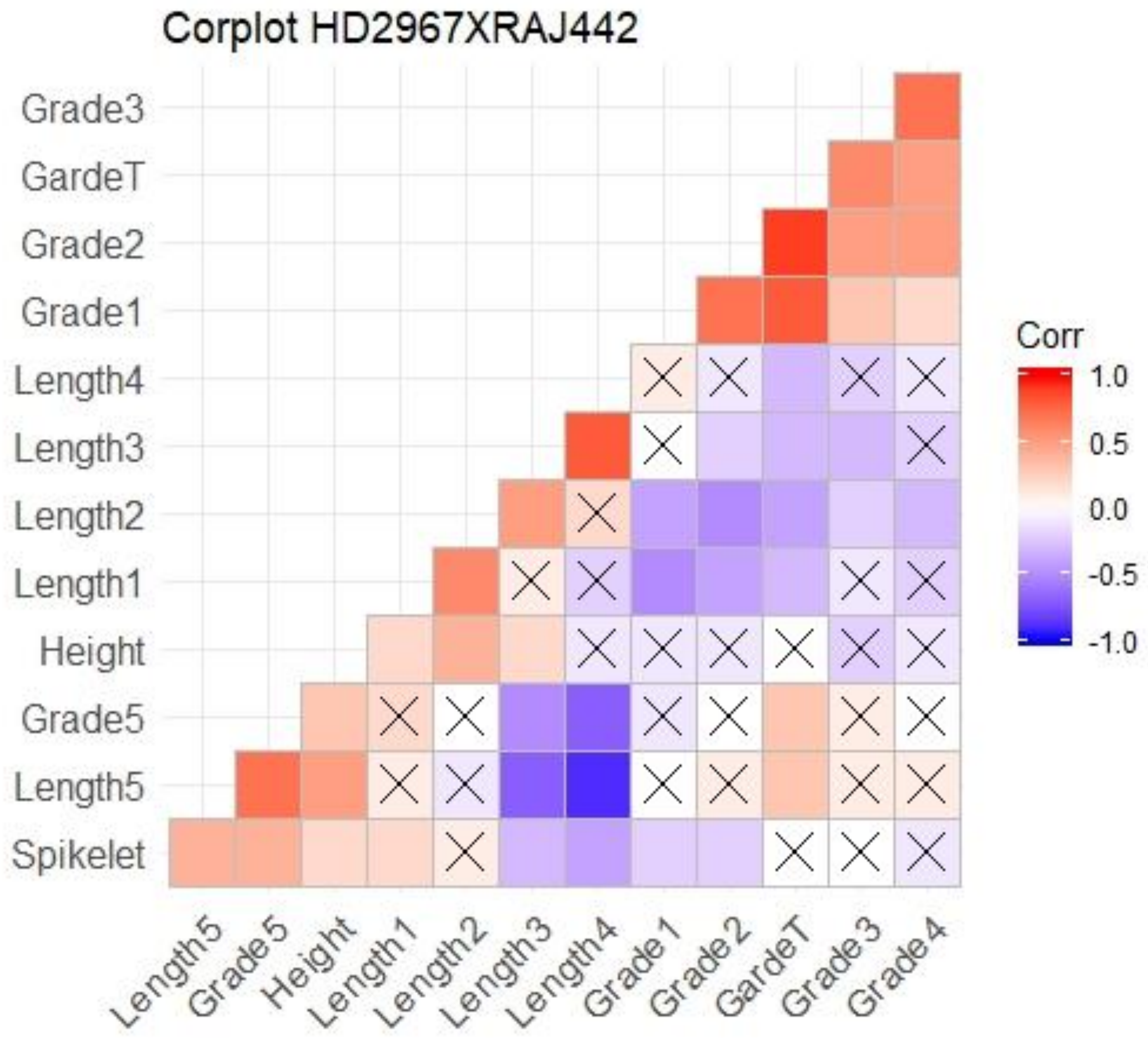

Figure 5.3 HD2967 × RAJ4422
Hierarchical clustering and correlation among Internode lengths, height and solidness grades

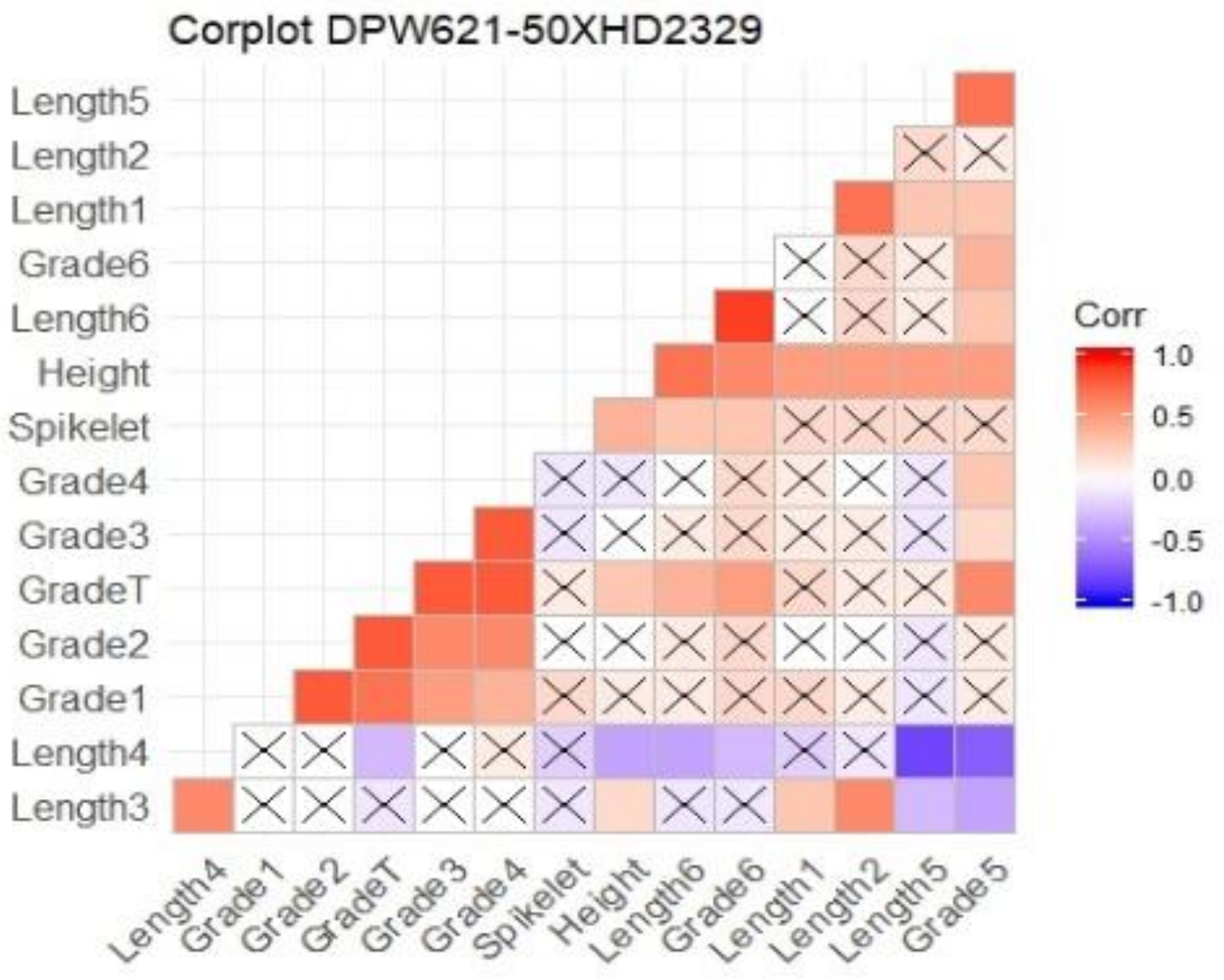

Figure 5.4 DPW621-50 × HD2189

Hierarchical clustering and correlation among Internode lengths, height and solidness grades

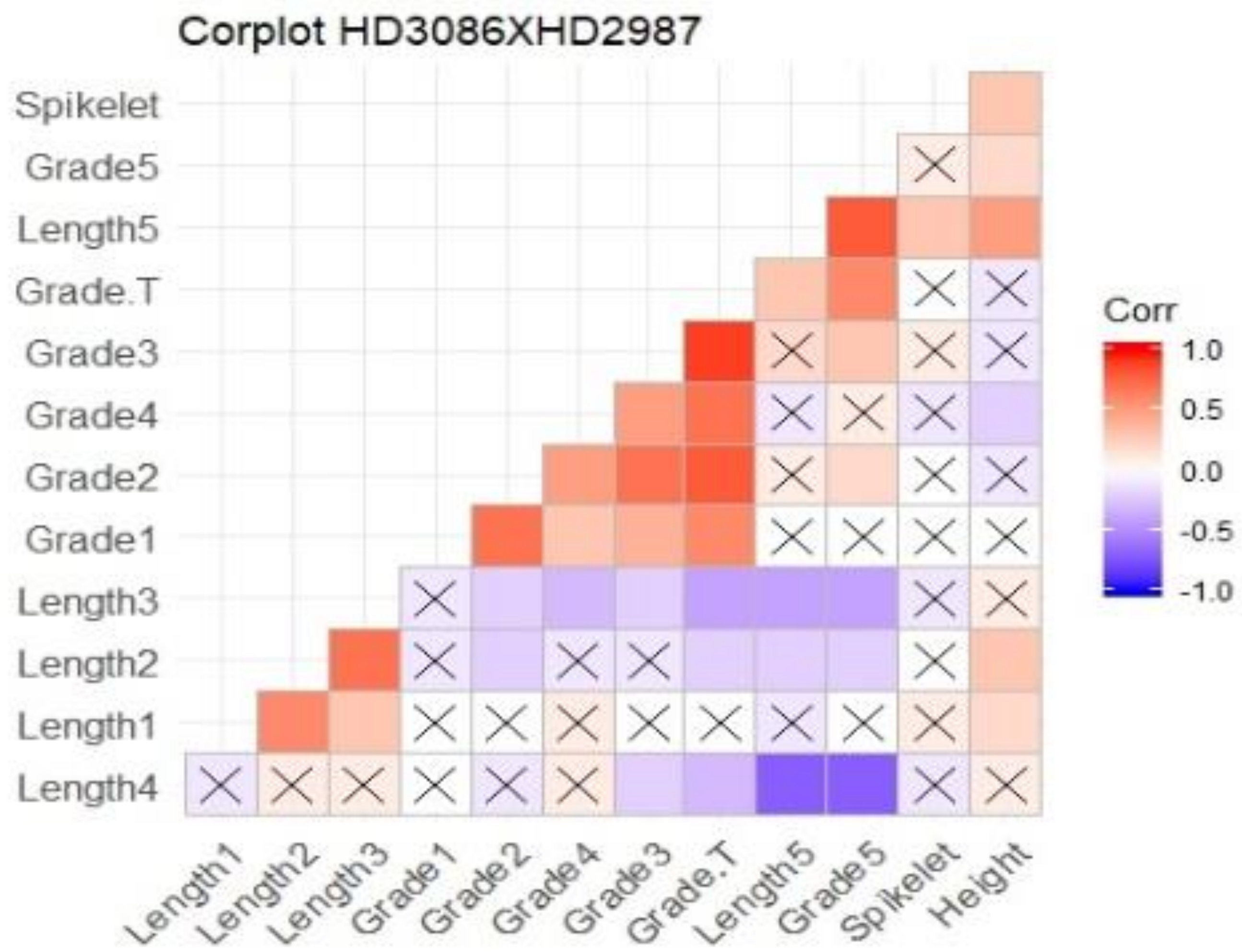

Figure 5.5 HD3086 × HD2987

Hierarchical clustering and correlation among Internode lengths, height and solidness grades

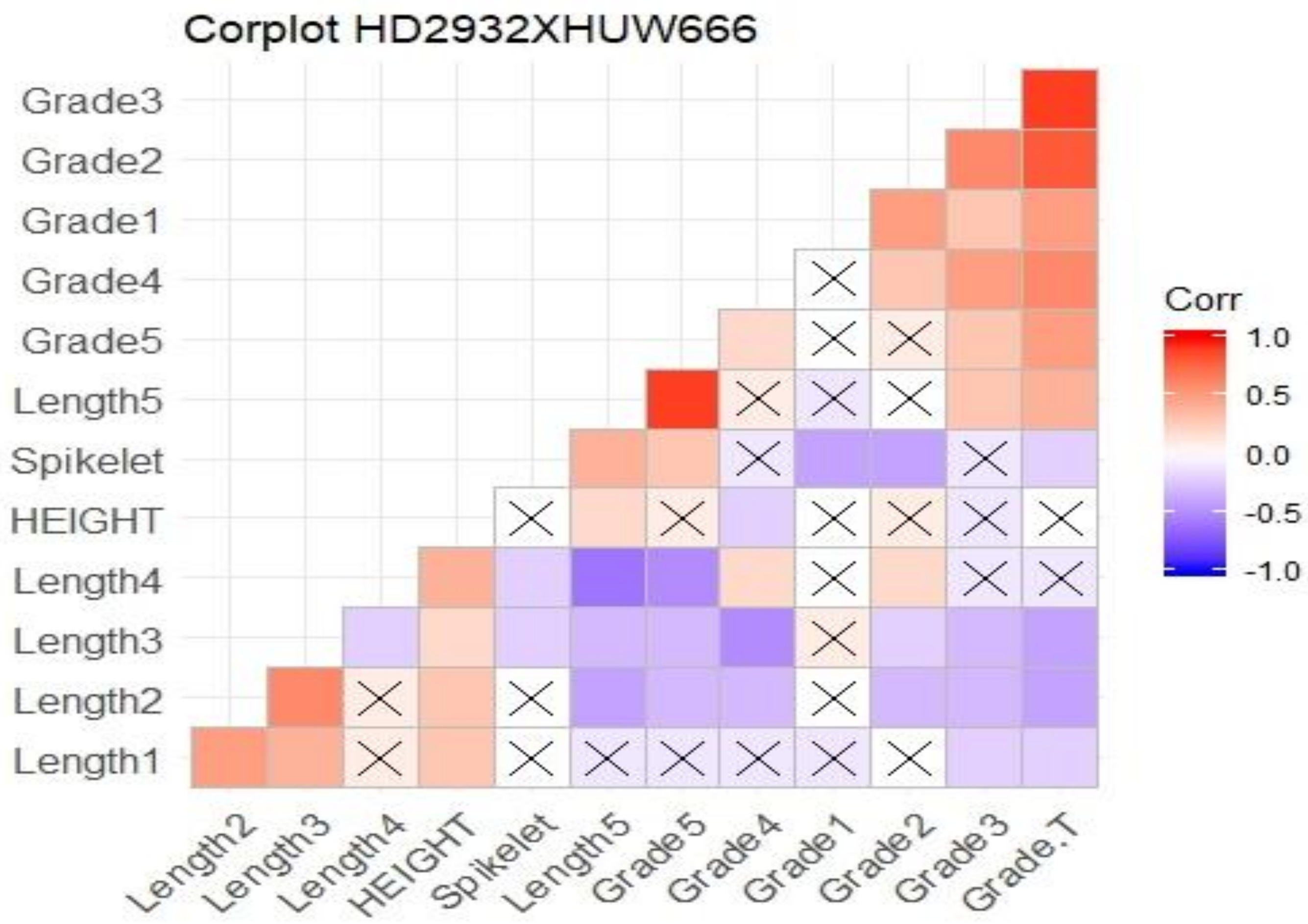

Figure 5.6 HD2932 × HUW666

Hierarchical clustering and correlation among Internode lengths, height and solidness grades

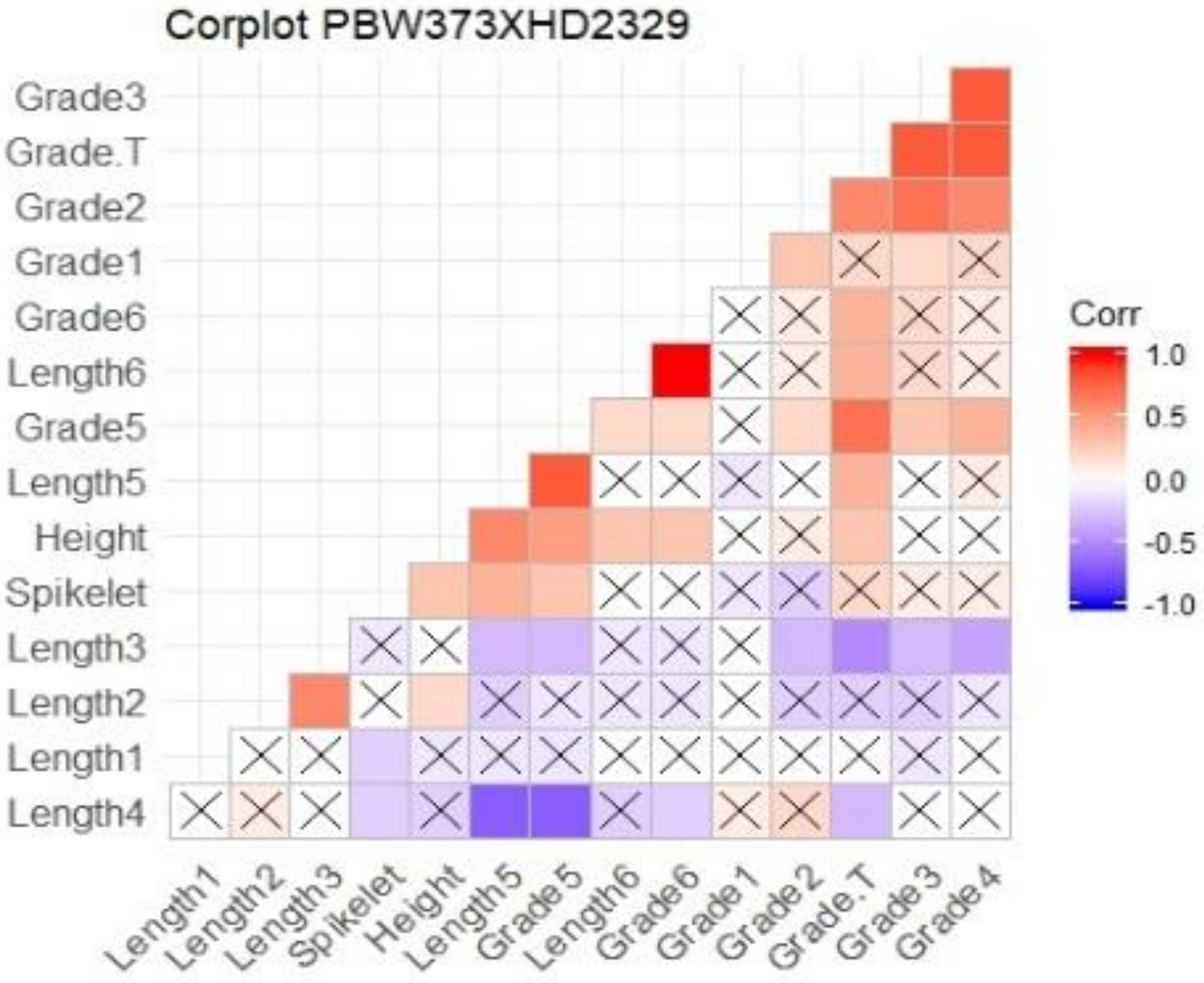

Figure 5.7 PBW373 × HD2329
Hierarchical clustering and correlation among Internode lengths, height and solidness grades

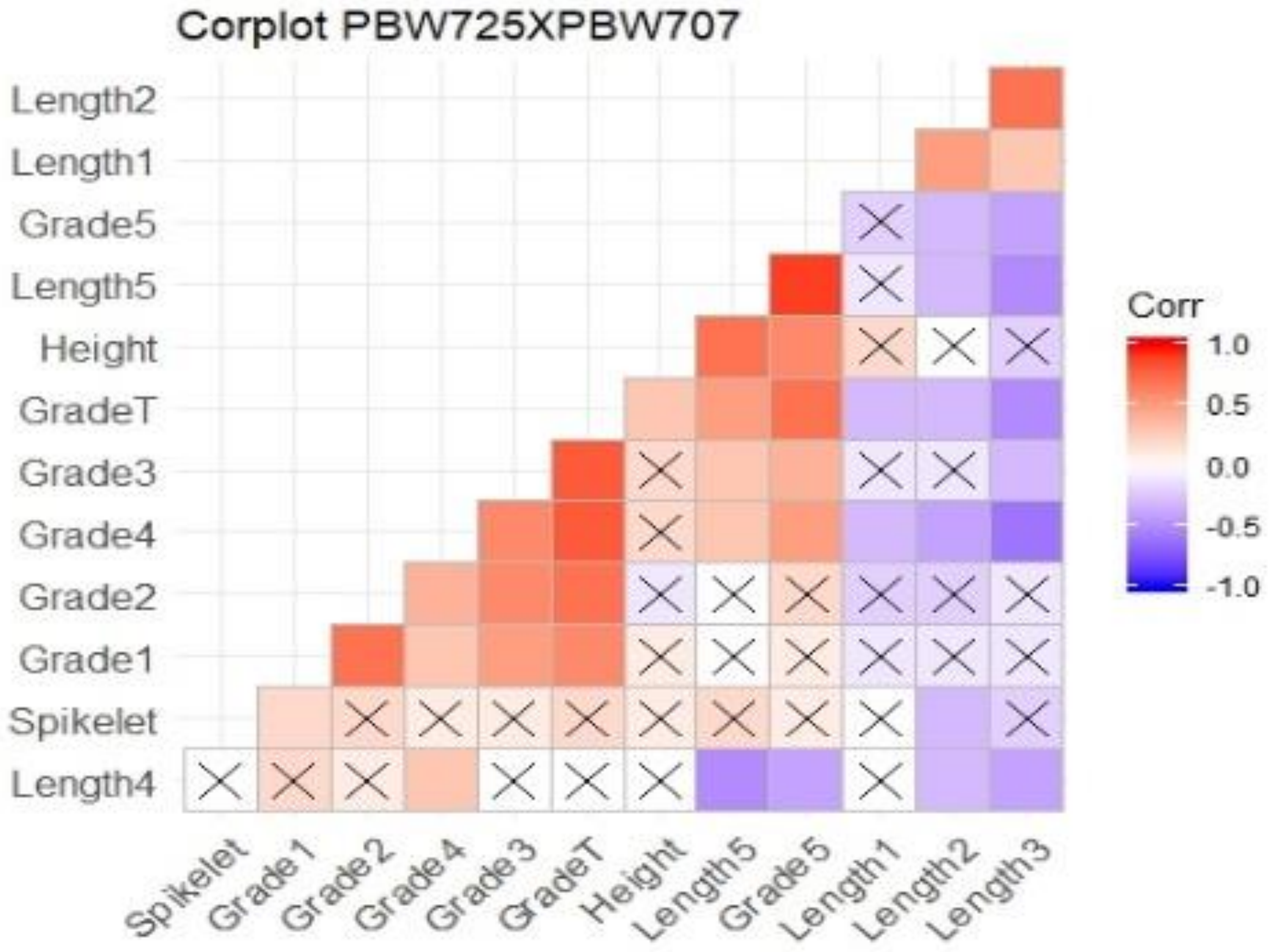

Figure 5.8 PBW725 × PBW707

Hierarchical clustering and correlation among Internode lengths, height and solidness grades

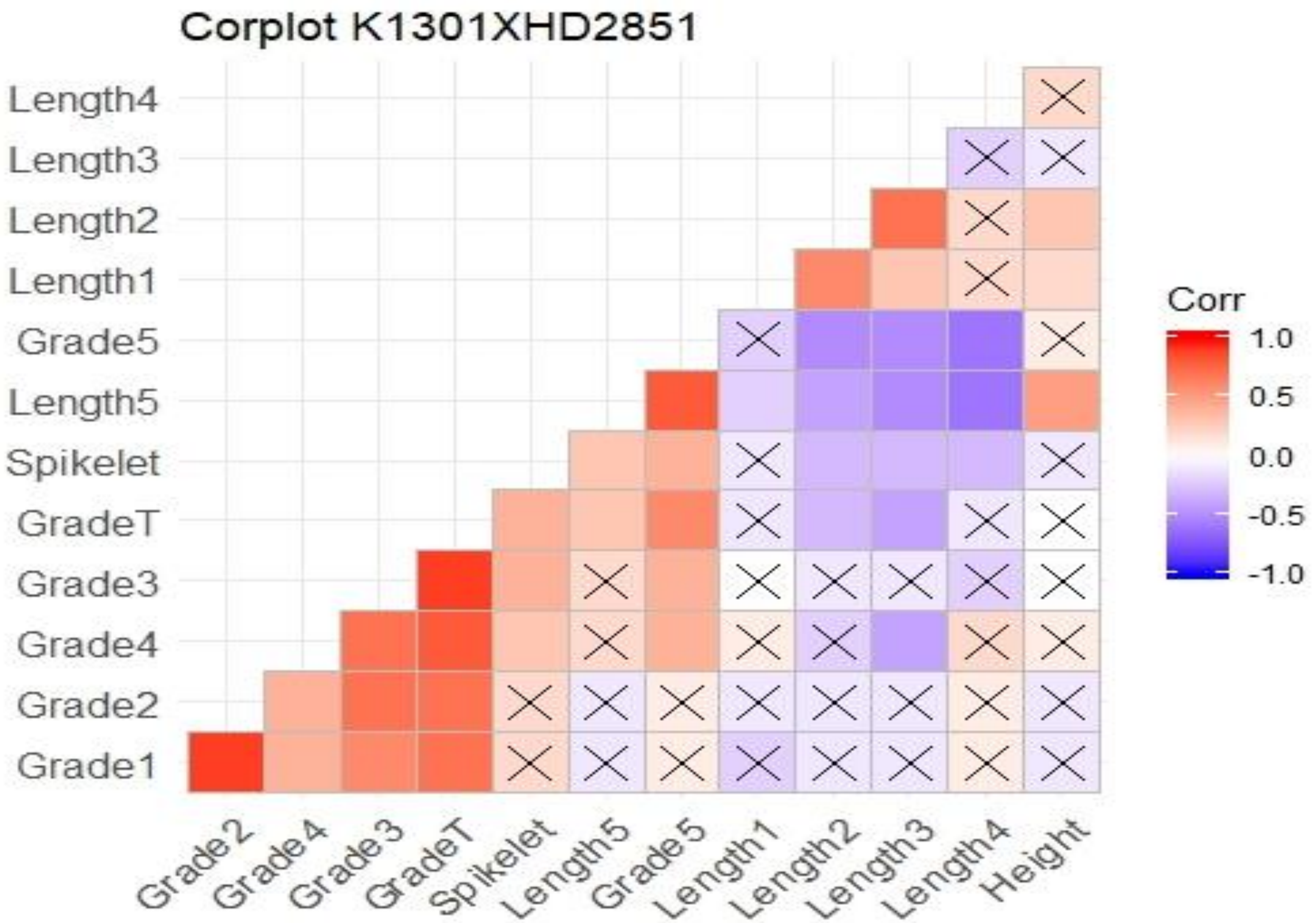

Figure 5.9 K1301 × HD2851/Yr10

Hierarchical clustering and correlation among Internode lengths, height and solidness grades

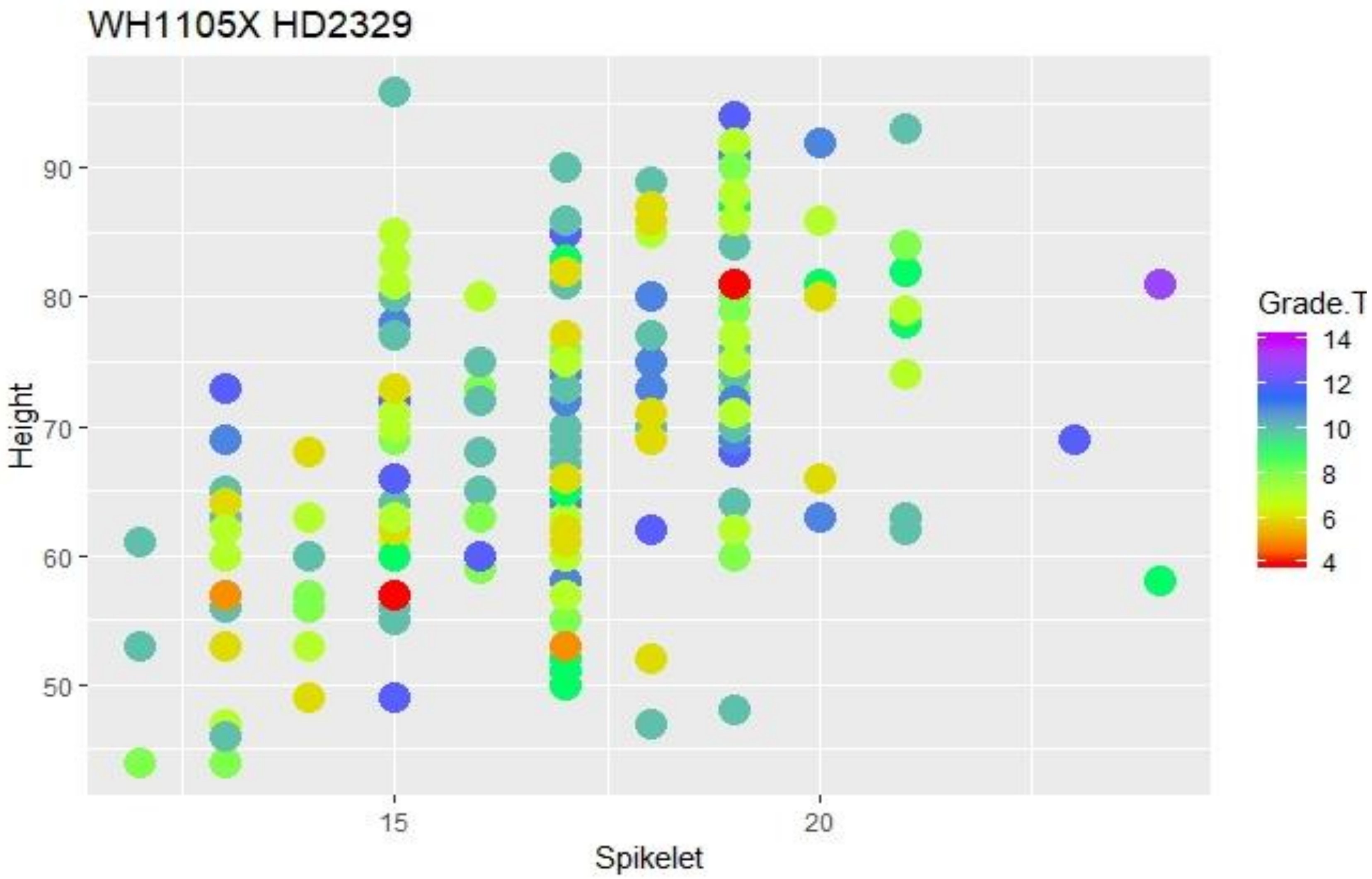

Figure 6.1 WH1105× HD2329
Graphical visualization for identification of desired recombinants (violet, blue and dark green) the individuals with solidness grade total of ≥8, spikelet number ≥20 and height ≥90cm.

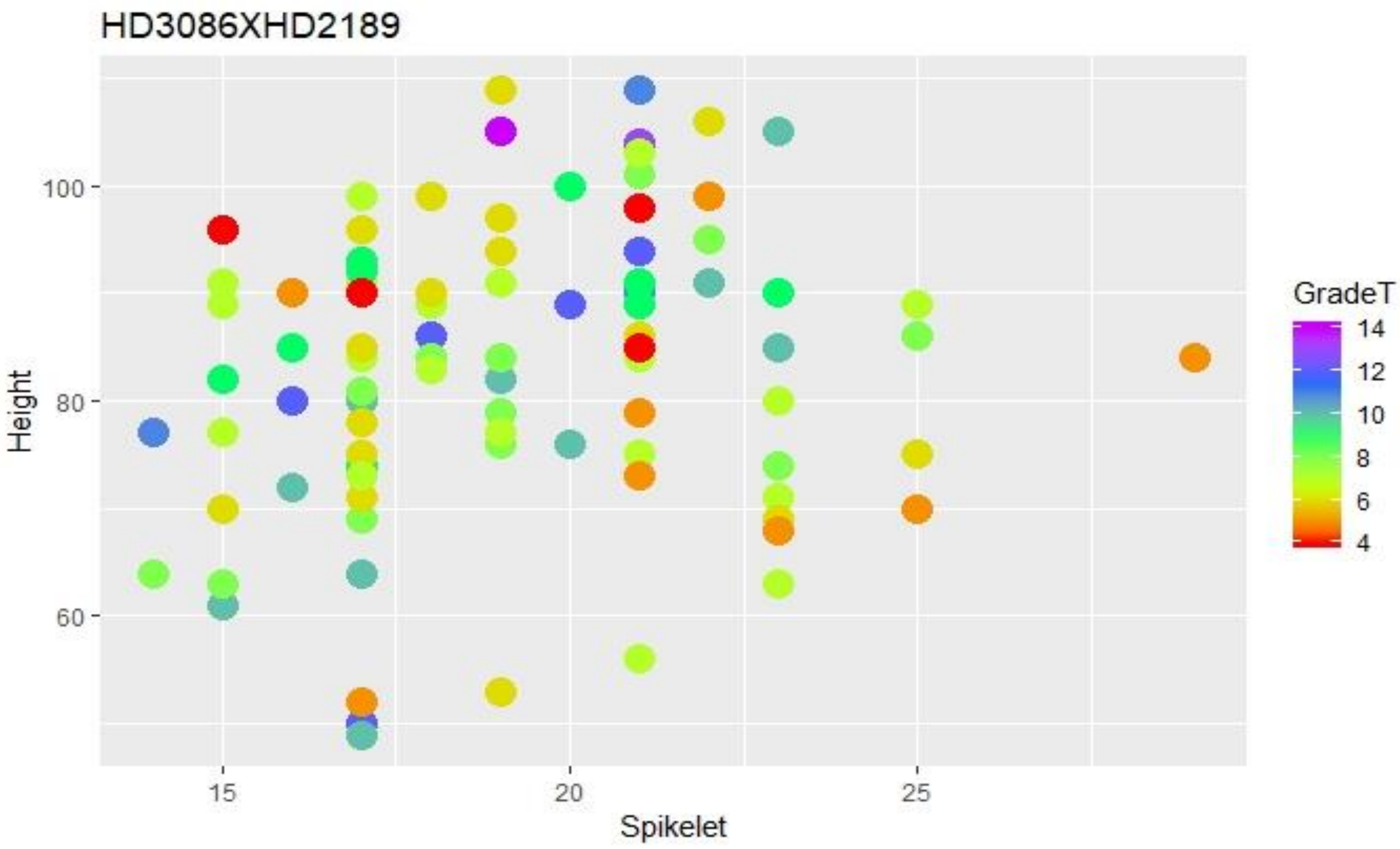

Figure 6.2 HD3086 × HD2189

Graphical visualization for identification of desired recombinants (violet, blue and dark green) the individuals with solidness grade total of ≥8, spikelet number ≥20 and height ≥90cm.

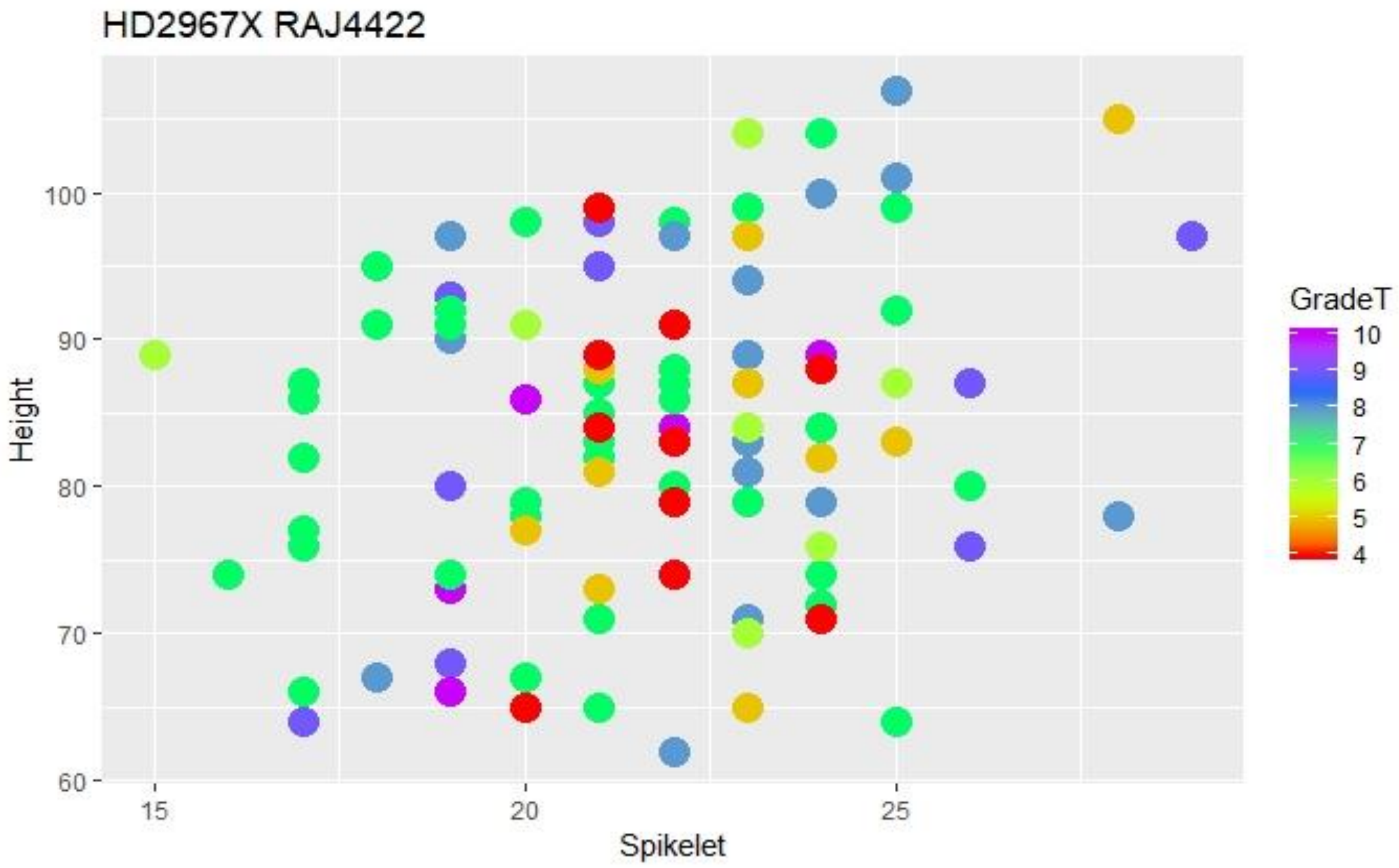

Figure 6.3 HD2967 × RAJ4422
Graphical visualization for identification of desired recombinants (violet, blue and dark green) the individuals with solidness grade total of ≥8, spikelet number ≥20 and height ≥90cm.

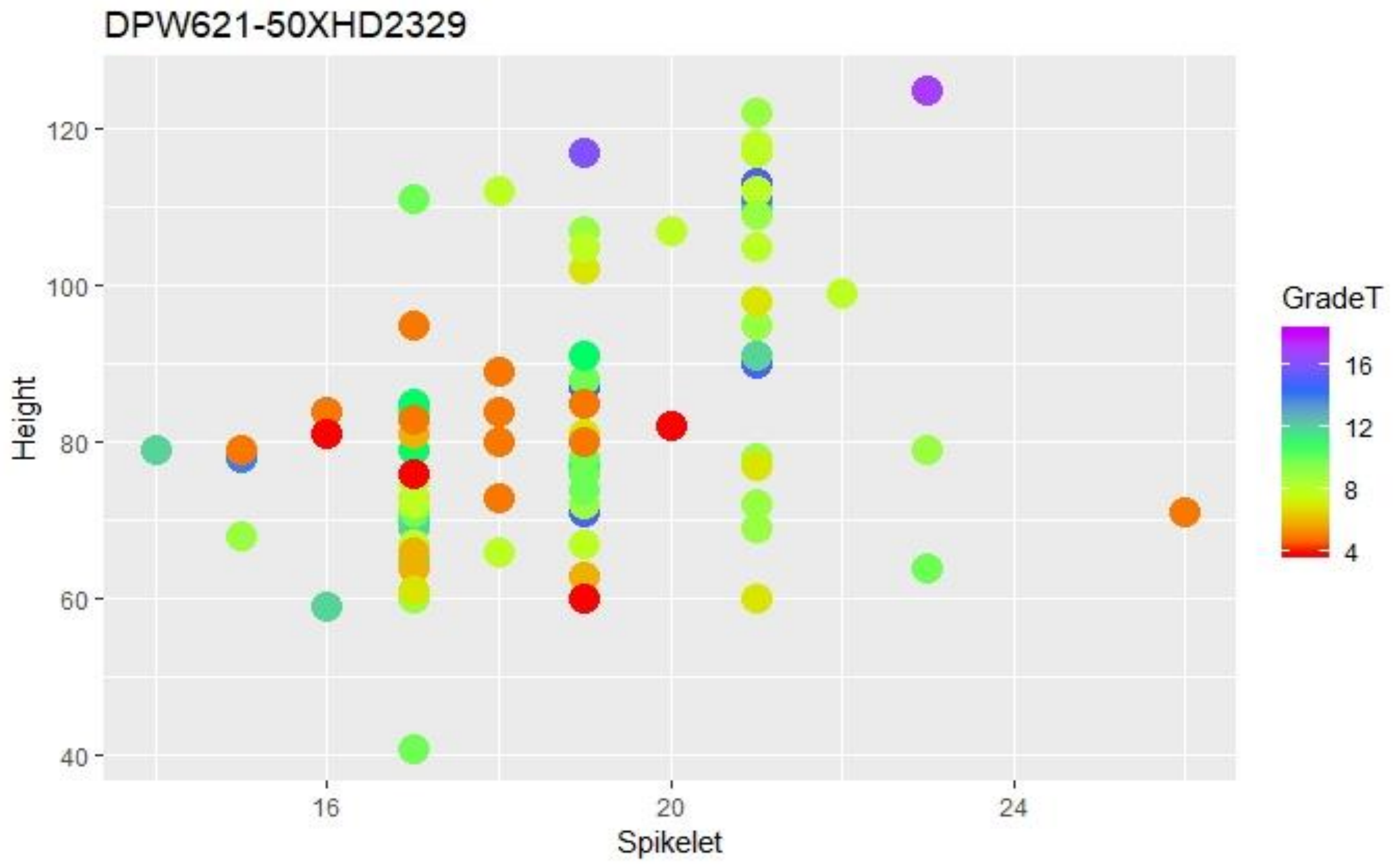

Figure 6.4 DPW621-50 × HD2189
Graphical visualization for identification of desired recombinants (violet, blue and dark green) the individuals with solidness grade total of ≥8, spikelet number ≥20 and height ≥90cm.

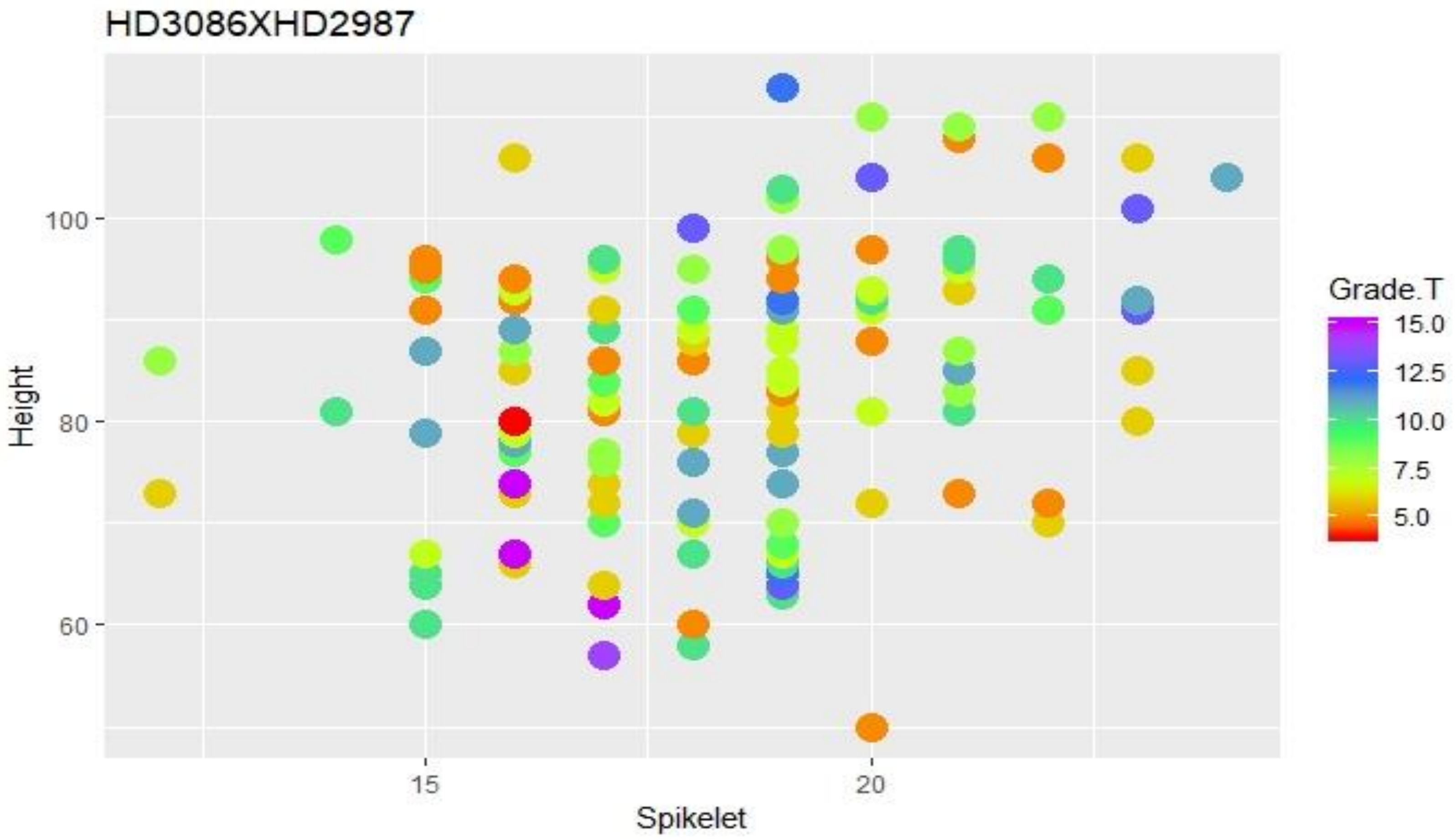

Figure 6.5 HD3086 × HD2987
Graphical visualization for identification of desired recombinants (violet, blue and dark green) the individuals with solidness grade total of ≥8, spikelet number ≥20 and height ≥90cm.

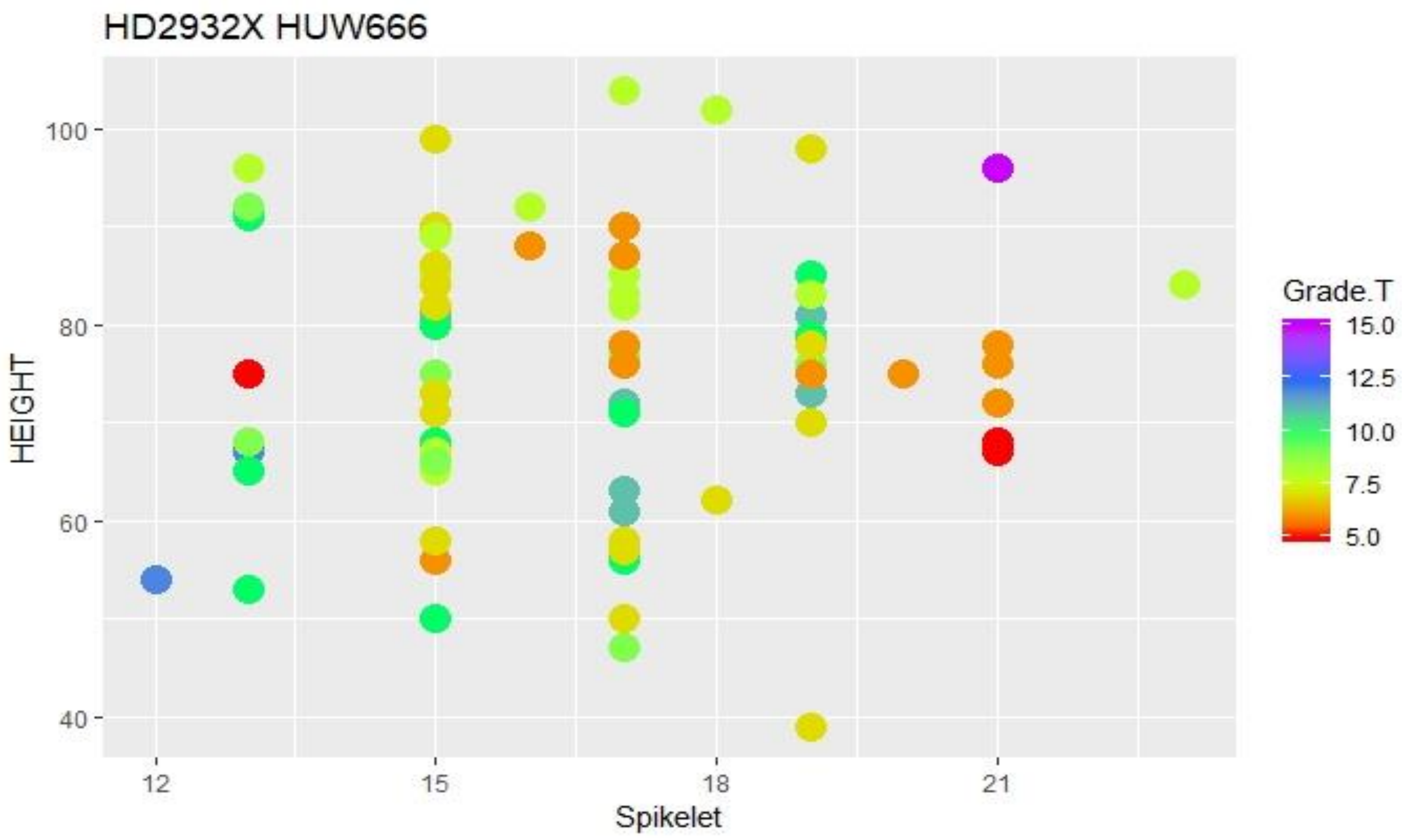

Figure 6.6 HD2932 × HUW666
Graphical visualization for identification of desired recombinants (violet, blue and dark green) the individuals with solidness grade total of ≥8, spikelet number ≥20 and height ≥90cm.

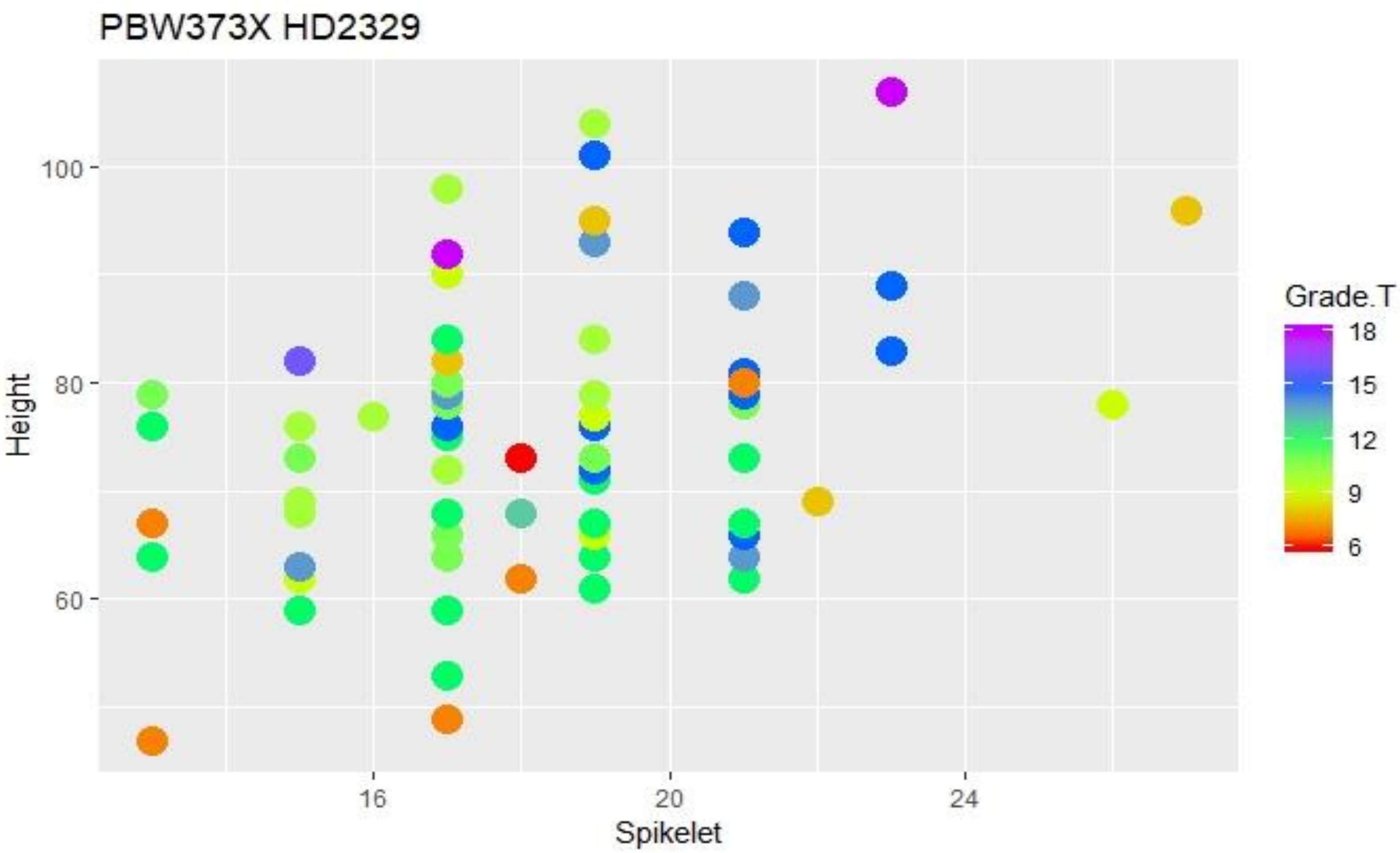

Figure 6.7 PBW373 × HD2329
Graphical visualization for identification of desired recombinants (violet, blue and dark green) the individuals with solidness grade total of ≥8, spikelet number ≥20 and height ≥90cm.

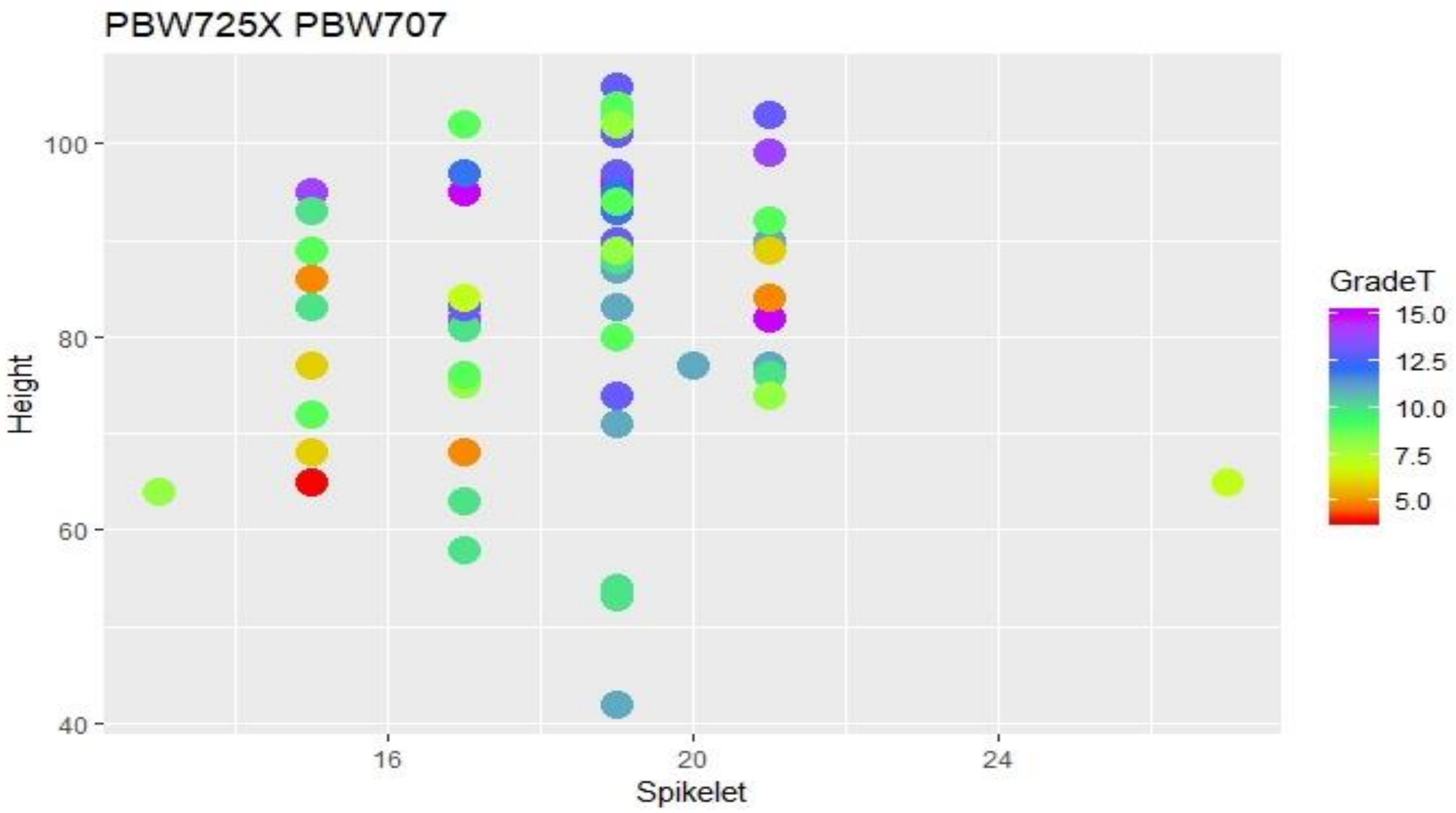

Figure 6.8 PBW725 × PBW707
Graphical visualization for identification of desired recombinants (violet, blue and dark green) the individuals with solidness grade total of ≥8, spikelet number ≥20 and height ≥90cm.

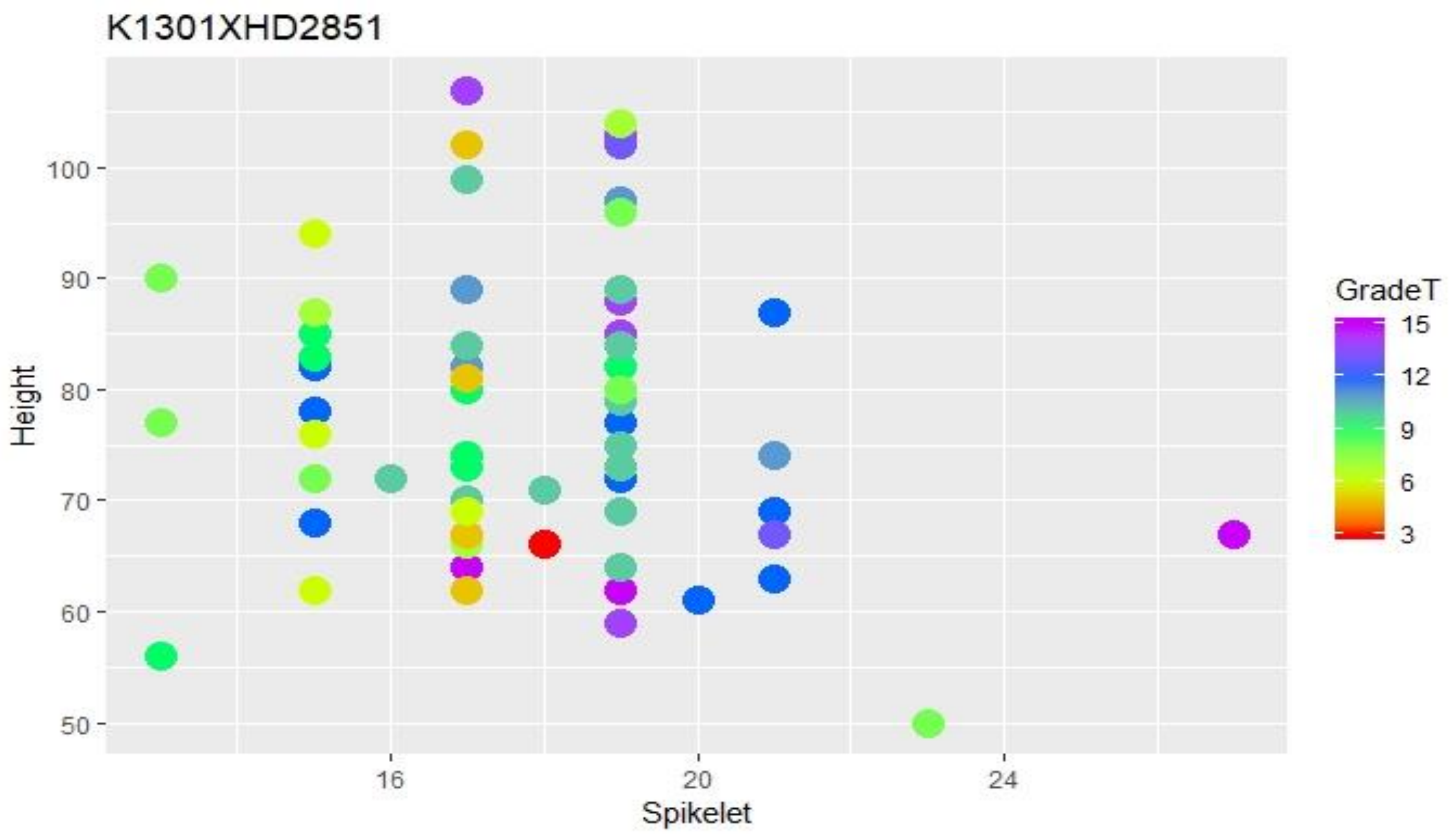

Figure 6.9 K1301 × HD2851/Yr10
Graphical visualization for identification of desired recombinants (violet, blue and dark green) the individuals with solidness grade total of ≥8, spikelet number ≥20 and height ≥90cm.

Pith expression level	Grade assigned	Category	Picture
<50% filled	1	Hollow	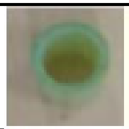
50-90% filled	2	Semi Solid	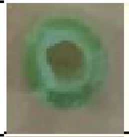
90-100%	3	Solid	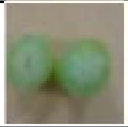

Supplementary Figure 7. Grades of solidness
